# Global progress and future prospects of early gastric cancer screening

**DOI:** 10.7150/jca.95311

**Published:** 2024-04-08

**Authors:** Yixiao Huang, Yongfu Shao, Xuan Yu, Chujia Chen, Junming Guo, Guoliang Ye

**Affiliations:** 1 Department of Gastroenterology, the First Affiliated Hospital of Ningbo University, Ningbo 315020, China.; 2 Department of Biochemistry and Molecular Biology, School of Basic Medical Sciences, Health Science Center, Ningbo University, Ningbo 315211, China.; 3 Institute of Digestive Disease of Ningbo University, Ningbo 315020, China.

**Keywords:** Gastric cancer, Early screening, Imaging, Endoscopy, Biomarkers, Noncoding RNA

## Abstract

Gastric cancer is a prevalent malignancy that poses a serious threat to global health. Despite advances in medical technologies, screening methods, and public awareness, gastric cancer remains a significant cause of morbidity and mortality worldwide. Early gastric cancer frequently does not present with characteristic symptoms, while advanced stage disease is characterized by a dismal prognosis. As such, early screening in gastric cancer is of great importance. In recent years, advances have been made globally in both clinical and basic research for the screening of early gastric cancer. The current predominant screening methods for early gastric cancer include imaging screening, endoscopic screening and serum biomarker screening. Imaging screening encompasses upper gastrointestinal barium meal, multidimensional spiral computed tomography (MDCT), Magnetic resonance imaging (MRI), and ultrasonography. Endoscopic screening methods include white light endoscopy, chromoendoscopy, computed virtual chromoendoscopy, and other endoscopic techniques like endocytoscopy, confocal laser endomicroscopy, optical coherence tomography and so on. Biomarkers screening involves the assessment of conventional biomarkers such as CEA, CA19-9 and CA72-4 as well as more emerging biomarkers such as peptides (PG, G-17, GCAA, TAAs and others), DNA (cfDNA, DNA methylation, MSI), noncoding RNA (miRNA, lncRNA, circRNA, and tsRNA) and others. Each screening method has its strengths and limitations. This article systematically summarizes worldwide progress and future development of early gastric cancer screening methods to provide new perspectives and approaches for early diagnostic and treatment advancements in gastric cancer worldwide.

## Introduction

**Figure 1:**
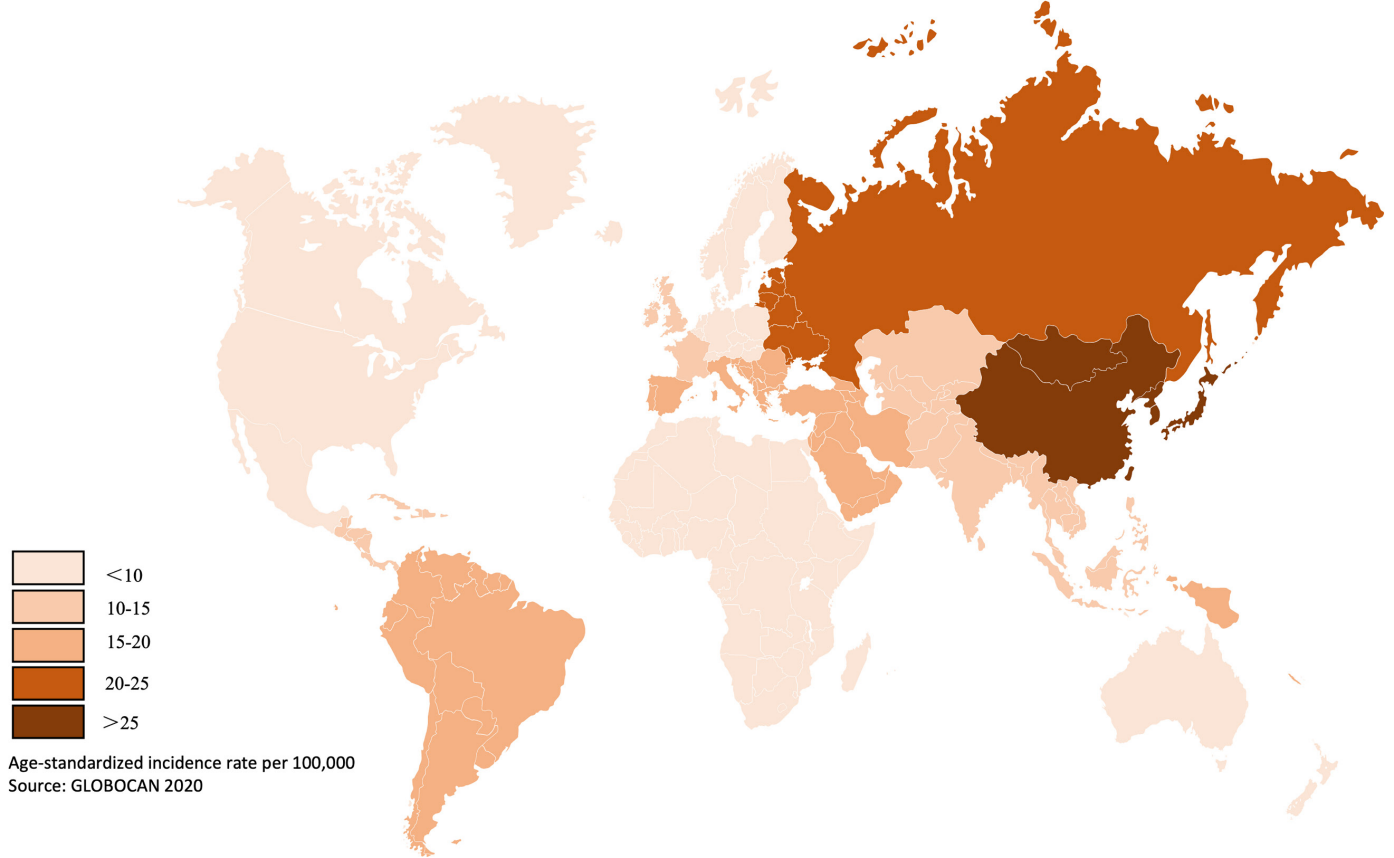
Global incidence rate of gastric cancer. The incidence varies globally, especially high in East Asia and east Europe. Data from published literature 1-5.

Gastric cancer is a prevalent gastrointestinal tumor, with the fifth and third highest incidence and mortality rates for malignant tumors, respectively 1. Objective statistics indicate that over 1 million cases were recorded in 2020 worldwide, resulting in over 768,000 fatalities 2. The incidence of gastric cancer is particularly high in East Asia, where it reaches up to 45.7 per 100,000 people (Figure 1) 2. In recent years, gastric cancer has remained a significant challenge for society, despite advancements in diet, hygiene and medical technology for screening and diagnosis.

Currently, the rate of early gastric cancer (EGC) detection varies globally. In Japan and Korea, over 50% of cases are detected early, while in Western countries such as the United States, the rate is only approximately 20% 4. In China, although its incidence accounts for 44.21% of the total number of cases worldwide, the rate of early gastric cancer detection is nearly 10~20% 3,5. Early gastric cancer typically lacks clear symptoms. By the time symptoms present, the cancer is often in the advanced stage, which can cause patients to miss the best opportunity for surgical intervention. For progressive gastric cancer, the primary treatment is the combination of neoadjuvant chemotherapy, molecular targeted therapy and immunotherapy 6. Despite the combined use of these therapies, the survival rate and prognosis of gastric cancer patients, particularly those with progressive gastric cancer, remain unsatisfactory. The survival rate of those with progressive gastric cancer is less than 30%, with the 5-year survival rate of stage IV gastric cancer patients being particularly low at less than 15% 7, 8. Conversely, the survival rate of patients with early gastric cancer is over 90% 7, 8. Thus, the primary strategy for enhancing the diagnosis and treatment of gastric cancer is early detection.

**Figure 2:**
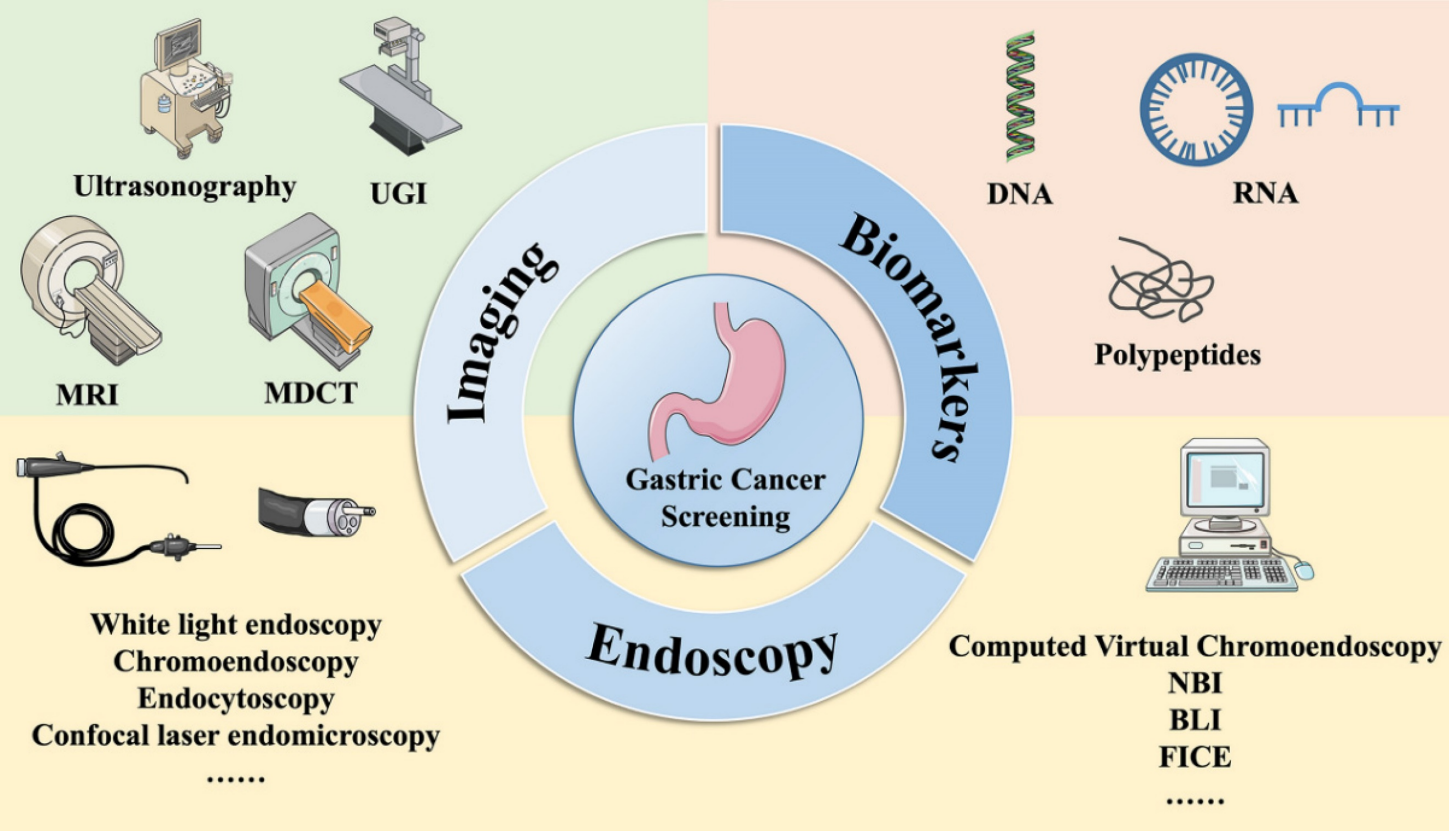
Methods of gastric cancer screening. Early gastric cancer screening methods usually involve imaging, endoscopy, and serum biomarker. UGI: Upper gastrointestinal barium meal; MRI: Magnetic resonance imaging; MDCT: Multi-dimensional spiral computed tomography; NBI: Narrow-band imaging; BLI: Blue laser imaging; LCI: Linked color imaging; FICE: Flexible spectral imaging color enhancement.

Studies have shown that screening for gastric cancer can substantially decrease mortality rates associated with the condition and enhance patient survival rates 9. Evidence from a range of countries and regions indicates that effective screening methods are available for the early detection of gastric cancer 10, 11. Early gastric cancer screening methods usually involve imaging, endoscopy, and biomarkers (Figure 2). Various imaging screening methods can be used to detect gastric disorders, such as upper gastrointestinal barium meal (UGI), multilayer spiral CT, upper abdominal MRI and gastric ultrasonography. Endoscopic techniques comprise white light endoscopy, chromoendoscopy, a variety of computed virtual chromoendoscopy strategies, such as narrow-band imaging (NBI) and blue laser imaging (BLI), cellular endoscopy, confocal laser microendoscopy, and optical coherence tomography, among other techniques. Biomarkers include conventional markers, specifically CEA, CA19-9, and CA72-4, which are commonly used in clinical practice as well as innovative biomarkers that include peptides (PG, G-17, GCAA, TAAs and others), DNA (cfDNA, DNA methylation, MSI), noncoding RNA (miRNA, lncRNA, circRNA, and tsRNA), and circulating tumor cells. This paper aims to provide an objective and concise summary of the current and potential advancements in early gastric cancer screening.

## 1. Imaging screening for EGC

**Figure 3:**
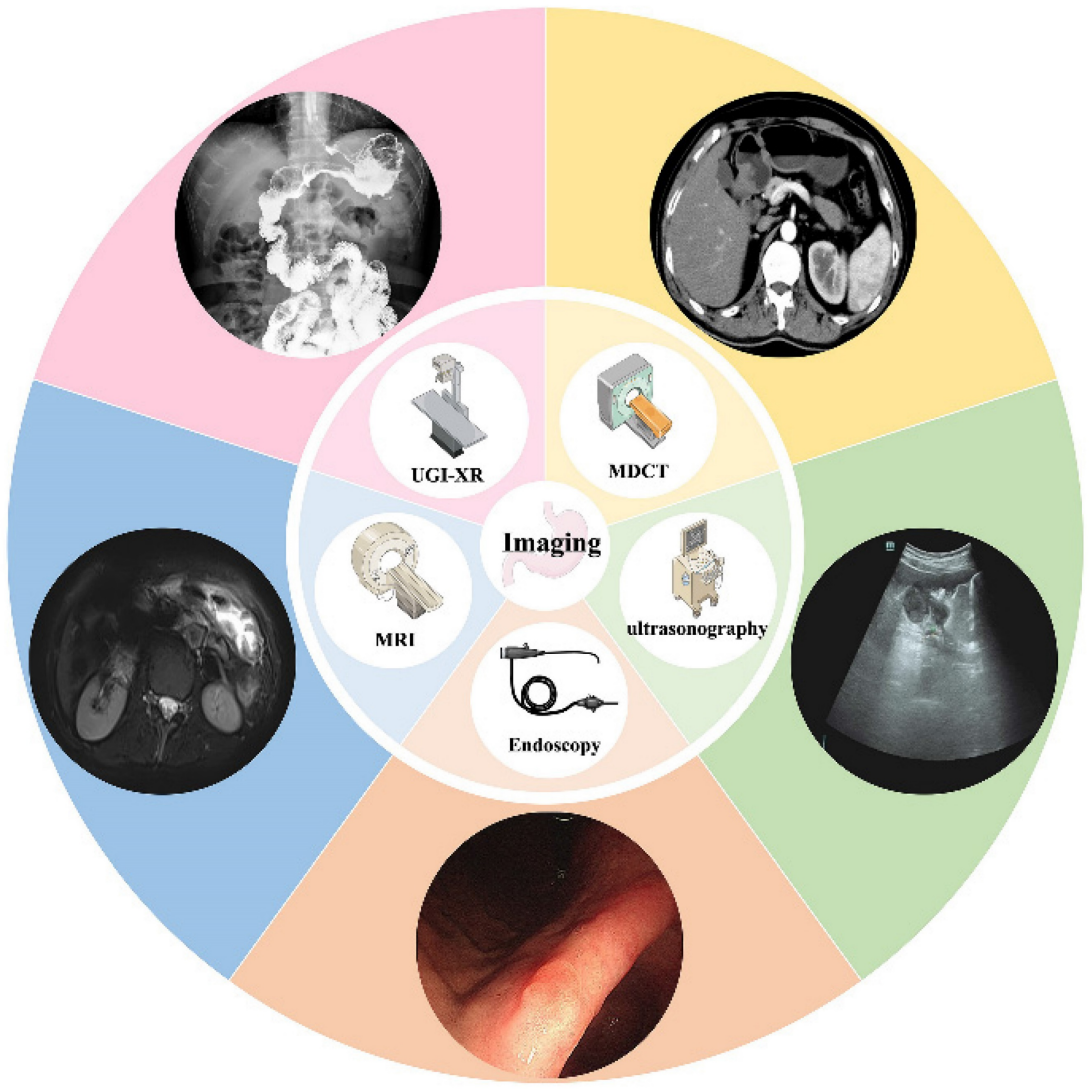
Imaging techniques of gastric cancer screening. Including UGI, MRI, gastric ultrasonography, and endoscopy.

Medical imaging plays a significant role in screening for gastric cancer due to its noninvasive, cost-effective and easily accessible features. From the classical upper gastrointestinal tract barium meal to modern-day multilayer spiral CT, MRI, and ultrasonography (Figure 3), advancements in equipment and techniques have greatly contributed to the detection of primary tumors, distant metastasis, and preoperative evaluation of gastric cancer.

### 1.1 Upper gastrointestinal barium meal

**Figure 4:**
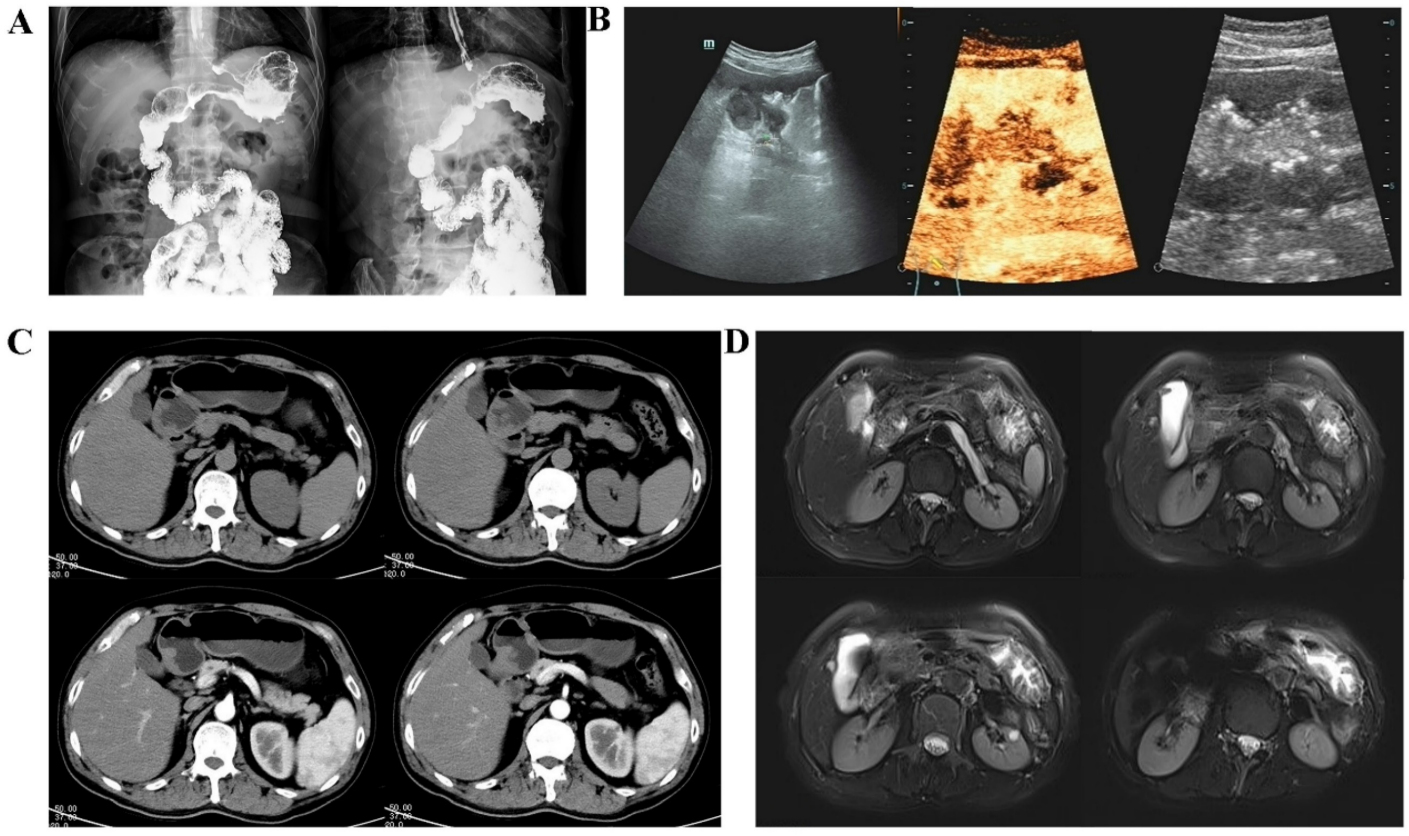
Typical images of imaging techniques of gastric cancer screening. (**A):** UGI: Localized rigidity of the gastric lumen, loss of normal mucosal morphology, and filling defects are observed. (**B):** Gastric ultrasonography: After oral administration of gastric window contrast agent, there are moderate echoes in the stomach, low echoes in the body of stomach, involving the body of stomach for one week, the range is about 116 × 41 mm, and local ulcers were found. Several hypoechoic lesions are found in the margin of the lesion, and the largest one is about 15 × 6 mm in size.** (C):** MDCT: The stomach is well filled, and the gastric wall of the gastric sinus is uneven and slightly thickened. After strengthening, it is slightly strengthened. (**D):** MRI: The mucosa of the greater curvature of the gastric body seemed to be thickened slightly and showed high signal intensity on DWI.

UGI is a conventional method of gastric cancer screening. This technique utilizes barium sulfate to distinguish lesions in the gastrointestinal tract under X-ray exposure (Figure 4A) 12.

In the 1980s, the Japanese Health Care Law integrated a gastric cancer screening program, which screened over 5 million individuals in one year 13. Of those screened, over 6,000 cases of gastric cancer were detected, with an overall detection rate of 0.12% 13. Early gastric cancer accounted for 50% of these cases, and the sensitivity, specificity, and positive predictive value were 82.4%, 77.2%, and 1.78%, respectively 13. With the arrival of double-contrast upper gastrointestinal barium radiography (UGI-XR), its diagnostic accuracy has improved even further 14, 15. Adequate cancer screening by UGI-XR has been demonstrated to decrease the risk of death caused by gastric cancer 16.

Although UGI is easy to perform, inexpensive and painless, its limitations have drawn public attention. Scholars have analyzed the reasons for its missed diagnoses and concluded that multiple factors, such as gastric lumen filling, barium concentration, operator's level, and gastric cancer morphology, affect its detection rate 17, 18. Additionally, it cannot reflect the depth of invasion of the lesion or be used to diagnose the involvement of neighboring organs and surrounding lymph nodes. Hence, its restricted usefulness in gastric cancer screening curtails its significance. The practice of "barium meal followed by endoscopy", which was established in the early years, has been progressively phased out 19.

### 1.2 Multidimensional spiral computed tomography

The most popular purpose of using multidimensional spiral computed tomography (MDCT) in gastric cancer is typically to determine the extent of local invasion and the existence or absence of distant metastasis 20. According to previous research, the T-staging accuracy in spiral CT scanning imaging is 84.5%, with that for the T1 stage even reaching 90.8% 21. Contrast-enhanced MDCT holds substantial benefits for the diagnosis of gastric cancer (Figure 4C) 22. In fact, some scholars have developed AI models to detect early gastric cancer (EGC) in CT portal-stage images in recent years 23.

There are two primary techniques for enhancing imaging clarity using either gas or water as the medium 24, 25. The former was demonstrated to be a good choice for detecting tumors and their relationship with the surrounding vascular structures of the gastric wall 25. The latter was found to be advantageous for evaluating the depth of infiltration of gastric cancers and in displaying tumor histological alterations. The detection rate of gastric cancer has been reported to be 65% (96.2% for advanced gastric cancer and 41.2% for early gastric cancer) 24, 26.

Its diagnostic value for gastric cancer, particularly for early gastric cancer and precancerous lesions, remains somewhat restricted. For instance, MDCT's utilization for diagnosing gastric cancer is based on the principle that a gastric wall thickness of greater than 1 mm may manifest as white bands or lines in the wall, while early-stage gastric cancer typically does not exhibit evident wall thickening 27. Moreover, the soft tissue contrast of CT images is relatively low.

### 1.3 Magnetic resonance imaging of the upper abdomen

Magnetic resonance imaging (MRI) offers superior soft tissue resolution to CT and has the ability to provide multiple sequences and contrast 28. In a clinical setting, MRI of the upper abdominal region is commonly utilized to assess solid organs, including the liver, gallbladder, and pancreas 29, 30. Currently, the principal method employed for gastric cancer screening is diffusion-weighted magnetic resonance imaging or DW-MRI (Figure 4D) 31.

In 2016, Tang and colleagues demonstrated the viability of DW-MRI for screening gastric cancers through a prospective study 32. In this study, four signal characteristics of gastric cancer were identified, and measurements revealed that the apparent diffusion coefficient (ADC) values were significantly lower in gastric cancer lesions than in normal gastric wall structures 32. Another retrospective analysis demonstrated that the precision and sensitivity of DW-MRI in identifying gastric cancer (77.8-78.3%; 75.3-75.9%) were substantially greater than those of CT (67.7-71.4%; 64.1-68.2%) or traditional MRI (72-73%; 68.8-70%) 33.

Nonetheless, DW-MRI is also subject to unavoidable limitations. The stomach, located in the epigastric cavity, is affected by both macroscopic motion and a low signal-to-noise ratio (SNR), leading to decreased examination accuracy 34, 35. Furthermore, DW-MRI is rather time-consuming and expensive.

### 1.4 Gastric ultrasonography

Gastric ultrasonography is a noninvasive, accurate, convenient and reproducible technique for detecting gastrointestinal diseases. In the fasting state, the patient is given an oral dose of an echogenic contrast agent to fill the stomach cavity, thus eliminating the interference of gas and contents in the gastric cavity to improve the quality of the image, and the sonographer is able to observe the structure of the gastric wall and its lesions in full-layer visualization, three dimensions, and real-time dynamics (Figure 4B) 36.

Liu et al. found that ultrasonography has a satisfactory detection rate of gastric cancer and precancerous lesions, with a detection rate of 77% for T1 b stage gastric cancer, 67% for T1 a stage gastric cancer, and even 60% for high-grade intraepithelial neoplasia 37. In addition, ultrasonography can be helpful for tumor staging and presurgical preparation 38, 39.

Ultrasonography has relatively few contraindications. However, some studies have also shown that ultrasonography is more accurate and sensitive in advanced gastric cancer, with accuracies and sensitivities of 79.7% and 98.6% in the advanced group and only 38.7% and 61.2% in the early group 40. Besides, its detection is also affected by body size, abdominal contents, tumor size, and the operator's technique 41, 42. So that it is not widely used in gastric cancer screening, especially in Western countries, and no clear evidence of its value can be found.

## 2. Endoscopy screening for early gastric cancer

**Figure 5:**
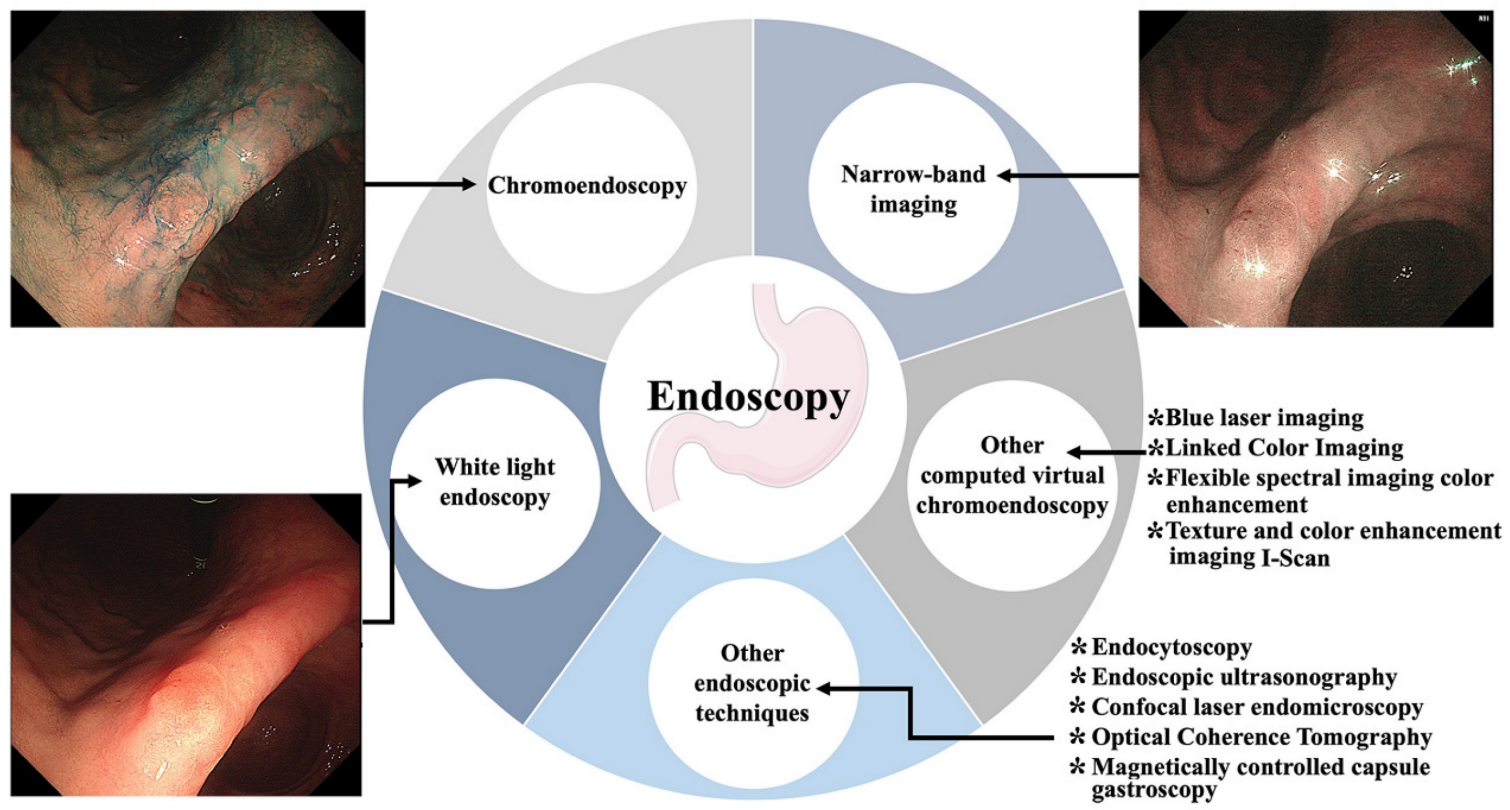
Endoscopy techniques of gastric cancer screening. Including white light endoscopy, chromoendoscopy, computed virtual chromoendoscopy and others.

The gold standard for diagnosing gastric cancer is "endoscopy + pathological biopsy". Scholars have been advancing science and technology to create endoscopic methods that are more convenient, fast, and accurate. As a result, chromoendoscopy, computed virtual chromoendoscopy and other technological means have emerged from white light endoscopy (Figure 5).

### 2.1 White light endoscopy

**Figure 6:**
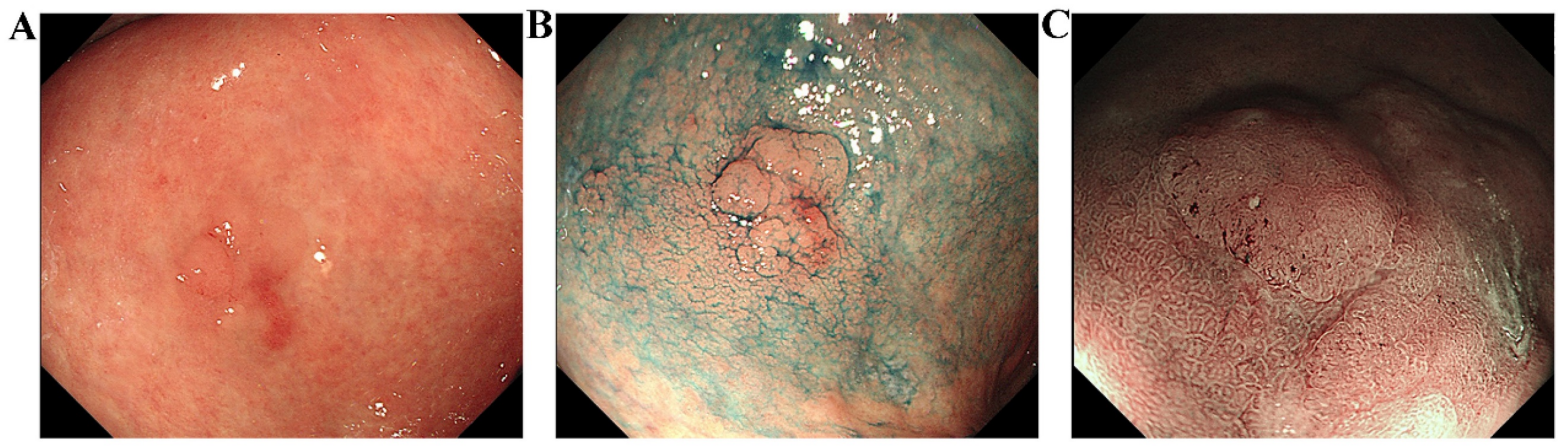
Images of a typical lesion of early gastric cancer. These endoscopic images show gastric mucosal lesions from a 77-year-old male patient. The lesion was discovered during a physical examination despite the absence of symptoms. (**A):** White light endoscopy. The mucosa of the gastric antrum appears reddish-white with extensive thinning and roughening. Blood vessels are partially visible. The lesion, measuring approximately 1.8cm x 2.0cm, is located on the anterior wall of the stomach. (**B):** Chromoendoscopy. This technique revealed a clearer boundary and outline of the lesion compared to white light endoscopy. (**C):** Magnifying-narrow band imaging. Observation of the surface microstructure is allowed by using this method. Based on the VS classification system, the lesion's scoring results indicate DL (+), IMVP (+), and IMSP (+), suggesting that it may be an early gastric cancer.

White light endoscopy (WLE) is the most widely used method (Figure 6A). High-definition white light endoscopy (HD-WLE) is gradually being introduced as technology advances. This technique has greatly improved image quality and has a sensitivity of 74.6%, specificity of 94%, and accuracy of 88% for detecting gastric cancer and precancerous lesions such as intestinal epithelial hyperplasia and atypical hyperplasia 43.

However, under white light, detecting some early microscopic lesions remains challenging. Significant mucosal lesions, including nodular, elevated, or depressed structures, can be effectively observed. However, slight mucosal irregularities or atypical coloration may be overlooked due to their subtlety. Previously, the missed rate for these conditions has been reported to be 9.4% according to a meta-analysis 44.

### 2.2 Chromoendoscopy

Chromoendoscopy, also known as dye endoscopy, involves staining the mucosal lining of the gastrointestinal tract with pigments or reagents to enhance the direct naked-eye observation and diagnosis of lesions (Figure 6B) 45. The primary methods of staining include spraying and oral administration. Commonly used pigments consist of compound iodine solution, methylene blue, and indigo carmine, and the most widely adopted method is endoscopic spraying of acetic acid and indigo carmine. These pigments can penetrate between the crevices of the GI mucosa to effectively reveal the details of the lesions to the examiner 46, 47.

The role of chromoendoscopy in identifying gastric cancer or precancerous lesions and their margins has been proven in research 48, 49. A prospective study conducted in Korea also determined this. Interestingly, conventional endoscopy has inadequate diagnostic capabilities for identifying tumor boundaries, achieving only a 66% success rate. Chromoendoscopy improved the identification rate of tumor boundaries to 84.1% 49, with particularly notable results for differentiated carcinomas, reaching up to 89.9% 49.

Nevertheless, there are some limitations to chromoendoscopy, including issues with localized over- or under-dyeing, insufficient bowel preparation, and a need for a longer examination time, all of which reduce its effectiveness to some degree 50. Furthermore, Lee et al. discovered that chromoendoscopy enhanced visibility of the horizontal margins of differentiated gastric cancers 49. Moreover, it did not yield satisfactory results with undifferentiated cancers such as imprinted cell carcinoma 50.

### 2.3 Computed virtual chromoendoscopy

#### 2.3.1 Narrow-band imaging

The Narrow-band imaging (NBI) technique is a widely utilized form of computed virtual chromoendoscopy that employs two wavelengths of light, blue and green, which are filtered for observation. When surface capillaries absorb blue light, they appear as a teal hue, while deeper blood vessels absorb green light, resulting in a blue color 51. This high-contrast visualization of difficult-to-see vascular patterns assists in lesion detection.

A multicenter randomized controlled trial conducted in Japan showed that the positive predictive value (PPV) of NBI was significantly greater than that of WLI (13.5% and 20.9%) and that having a sufficiently high PPV lowered the need for needless biopsies and their corresponding bleeding risk 52. Additionally, the proportion of low-grade adenomas detected by NBI was higher than that detected by WLI (90.9% and 70.8%, respectively) 52. Ascribed to the vascular plus surface structure (VS) classification system, the amalgamation of NBI and magnifying endoscopy, M-NBI, has been denoted an "optical biopsy" (Figure 6C) 53. M-NBI has been shown to exhibit superior levels of accuracy (90.4% vs. 64.8%) and specificity (94.3% vs. 67.9%) compared to white light endoscopy 54.

However, M-NBI only enables visualization of the surface mucosal layer. It has been noted that the diagnostic challenges of M-NBI may be linked to histological characteristics, such as low tumor heterogeneity, superficial tumor layers that are covered by noncancerous epithelial cells, and similar surface structures between cancerous and noncancerous tissues 55. Furthermore, the superiority of NBI or even M-NBI in illustrating tumor margins, particularly in undifferentiated tumor types, has not been established 56, 57.

#### 2.3.2 Blue laser imaging

Blue laser imaging (BLI) is an endoscopic imaging technique that utilizes the property of hemoglobin to absorb short wavelengths and the reflection of light by mucous membranes for observation and diagnosis of surface micro vessels and deep blood vessels 58. The BLI-bright mode implements BLI with a reduced narrowband light component, enhancing image brightness. The detection rate of gastric cancer by this method has been shown to be considerably higher than that observed with WLI (93.1% vs. 50.0%) 59. Moreover, its benefits are especially apparent in patients with a history of endoscopic resection, *Helicobacter pylori* (*H. pylori*) eradication, open atrophic border lesions, lower third of the stomach lesions, and recessed lesions. Coupled with magnifying endoscopy, small distinctive alterations in gastric cancer are more distinctly visualized 60-62. Compared to WLI, M-BLI demonstrates a considerable enhancement in the diagnostic accuracy of both gastric cancer and precancerous lesions 60, 61, 63.

#### 2.3.3 Linked color imaging

Linked color imaging (LCI) includes a larger component of narrow-wavelength light that can be absorbed specifically by hemoglobin, facilitating the highlighting of microstructures and blood vessels on the mucosal surface and providing clear distance imaging without magnification 64, 65. A report highlighted that LCI has a significantly higher mean color difference compared to WLI [17.2 (5.9) vs. 10.1 (4.4)], depicting its effectiveness in highlighting mucosal changes 66. Several clinical studies have shown that LCI not only has a markedly lower rate of gastric cancer leakage than white light endoscopy 67, 68 but also is highly effective for post eradication imaging of *H. pylori* lesions and flat or indurated lesions 69. LCI has also been utilized for the diagnosis of gastritis, *H. pylori* infection, gastrointestinal epithelial hyperplasia, and early gastric cancer 64.

#### 2.3.4 Flexible spectral imaging color enhancement

As opposed to NBI, BLI, and other techniques that alter light composition, flexible spectral imaging color enhancement (FICE) employs software-driven image postprocessing. In FICE images without magnification, the median color difference between the malignant lesion and surrounding mucosa is more pronounced compared to conventional images, leading to better image contrast (27.2 versus 18.7) 70, 71. When combined with magnifying endoscopy (FICE-ME), FICE allows for the achievement of an "optical biopsy". This is helpful in determining the degree of differentiation of gastric cancer by generating a magnified microvascular pattern. Furthermore, FICE is useful in determining the extent of the lesion for complete resection of EGC by ESD 71.

#### 2.3.5 Texture and color enhancement imaging

Texture and color enhancement imaging (TXI) enhances three key imaging factors—texture, brightness and color—through the use of white light endoscopy to improve visibility 72. Several studies have demonstrated that the visibility of gastric cancer is significantly enhanced by TXI 73-75. Specifically, one study found that the color difference between early gastric cancer lesions and nontumor mucosa was significantly greater with TXI than with WLI (TXI: 16.0±10.1 vs. WLI: 10.2±5.5 [mean±standard deviation]) 73. Furthermore, TXI has been found to be effective in improving the visibility of EGCs following *H. pylori* eradication 76.

#### 2.3.6 I-Scan

I-Scan is a software-based digital technique. The algorithm processes white light images into surface enhancement (SE), contrast enhancement (CE), and tonal enhancement (TE) 77, 78. SE and CE mostly are used for detecting gastric mucosal lesions, when lesions are scrutinized after discovery, TE can be selected as needed 77, 78. Several studies have found that i-Scan does not provide significant advantages in detecting gastric cancer lesions or determining their extent when compared with high-definition white light endoscopy or pigmented endoscopy. However, the use of i-Scan could largely reduce the need for unnecessary biopsies compared to WLE (with a mean number of biopsies per patient of 3.27 and 7.3, respectively) 79, 80.

### 2.4 Other endoscopic techniques

#### 2.4.1 Endocytoscopy

Endocytoscopy (EC) is an endoscope featuring ultrahigh resolution and magnification capabilities that enable the detection of cellular and nuclear abnormalities, thereby presenting the potential to replace conventional histopathological biopsy 81. EC has been extensively used to diagnose benign and malignant gastric mucosa 82. A study reported that EC can be used to accurately diagnose gastric cancer using cellular anisotropy, with a sensitivity of 86%, specificity of 100%, positive predictive value of 100%, and negative predictive value of 94% 83.

#### 2.4.2 Endoscopic ultrasonography

Endoscopic ultrasonography (EUS) provides valuable information about lesion infiltration depth and is regarded as superior to tests such as MDCT in distinguishing between mucosal and submucosal lesions. However, its accuracy has shown a wide range, from 65.0% to 92.1%, and is significantly influenced by factors such as ulceration, lesion location, disease stage, and tumor size 84, 85.

#### 2.4.3 Confocal laser endomicroscopy

Confocal laser endomicroscopy (CLE) is a technique that combines confocal laser microscopy with conventional electron endoscopy 86. A meta-analysis systematically evaluated the diagnostic effectiveness of CLE and narrow-band imaging (NBI) in the diagnosis of focal precancerous stages of gastric cancer. The meta-analysis revealed that both CLE and NBI demonstrated excellent diagnostic efficiency, with the pooled sensitivity (90% vs. 87%) and specificity (87% vs. 85%) of CLE being slightly higher than those of NBI 87.

#### 2.4.4 Optical coherence tomography

Optical coherence tomography (OCT) is a 3D imaging technique that provides cross-sectional images of living tissue morphology with micron-level resolution. Its objective use extends to screening gastric cancer and precancerous lesions by distinguishing between polyp tissue, normal tissue, and malignant tissue 88.

#### 2.4.5 Magnetically controlled capsule gastroscopy

Magnetically controlled capsule gastroscopy (MCCG) enables accurate maneuvering of the capsule endoscope to any area within the stomach, regulated by an external magnetic field 89. This technique has the benefits of being noninvasive, pain-free, and not increasing the likelihood of cross-infection. Previous research has revealed that MCCG can be effective in screening for gastric cancer and precancerous conditions, with diagnostic sensitivity and accuracy rates sometimes exceeding 90% 90.

Furthermore, various novel methods are progressively utilized to diagnose gastric cancer and its precancerous lesions. These methods encompass red dichromatic imaging (RDI), autofluorescence endoscopy autofluorescence imaging (AFI), and others. Nonetheless, their efficacy in detecting early-stage gastric cancer demands further scrutiny. In recent years, artificial intelligence (AI) technology has become a key area of interest within the field of gastric cancer screening and digestive endoscopy. Previous research indicates that AI systems can enhance the detection accuracy of lesions, distinguish between cancerous and noncancerous lesions, describe lesion borders, detect their differentiation status, and anticipate the depth of invasion 91, 92. Improving the endoscopist's ability to recognize gastric cancer, applying suitable optical techniques promptly, and obtaining pathological biopsies upon detecting indeterminate lesions are crucial aspects for improving the diagnostic rate of gastric cancer and precancerous lesions, alongside the emerging technologies available.

## 3. Biomarker screening for early gastric cancer

**Figure 7:**
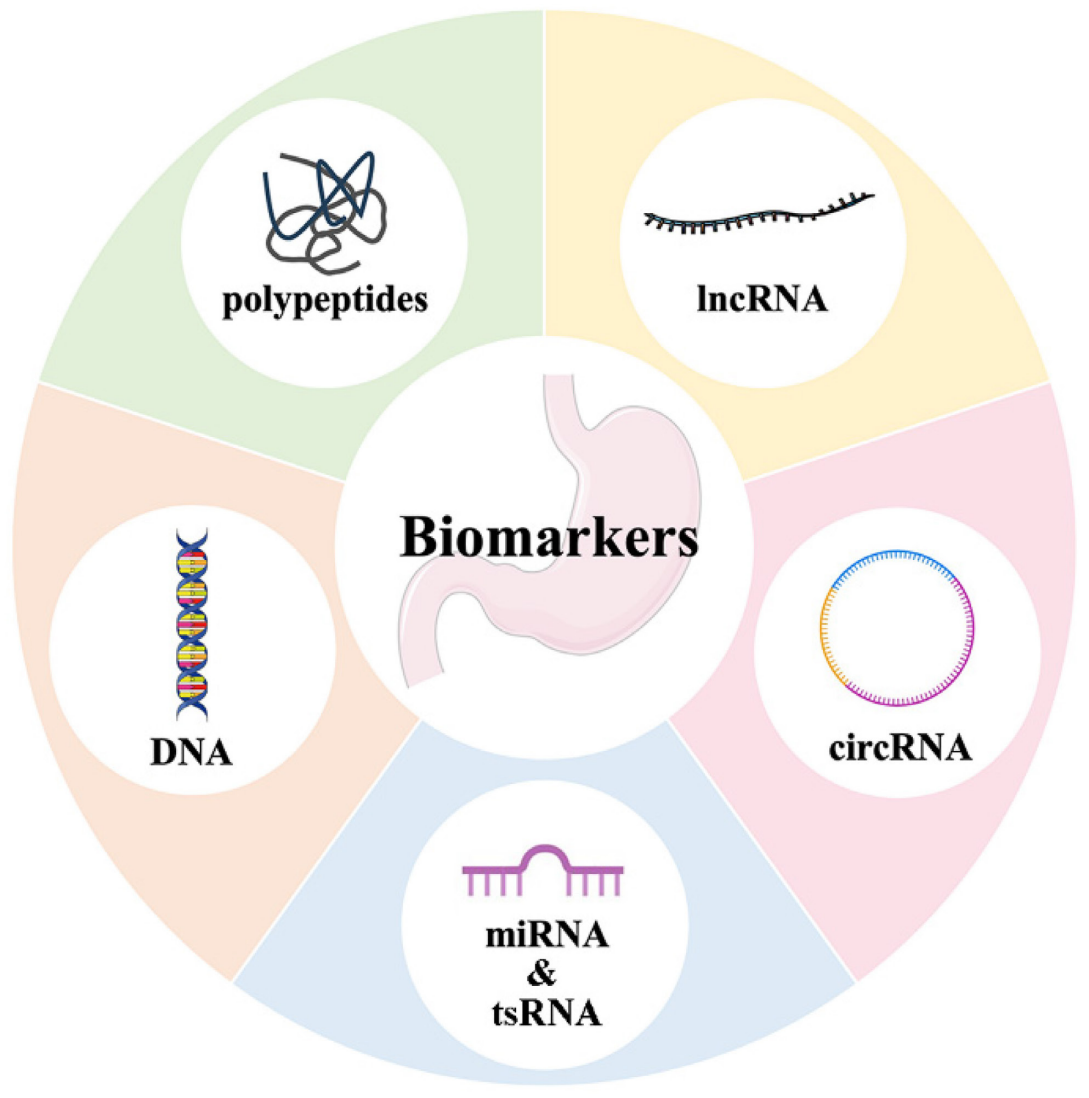
Biomarkers of gastric cancer screening. Including traditional biomarkers such as CEA, CA199, CA724, and novel biomarkers which are generally classified into four main groups: polypeptides, DNA, RNA, and other categories.

Biomarker screening is favored by patients undergoing gastric cancer screening due to their noninvasiveness, inexpensiveness, and ease of administration. The availability of biomarkers that offer high sensitivity and specificity greatly aids in the detection of early gastric cancer. Currently, apart from the conventional biomarkers of CEA, CA199, CA724, and others, there exist numerous biomarkers that have demonstrated greater reliability. These biomarkers are generally classified into four main groups: polypeptides, DNA, RNA, and other categories (Figure 7).

### 3.1 Traditional biomarkers

#### 3.1.1 Carcinoembryonic antigen

Carcinoembryonic antigen (CEA) is the most commonly used tumor marker in the gastrointestinal tract, with reported positive rates ranging from 4.3% to 25.5% 93, 94. However, it is widely accepted that CEA alone has low positivity and sensitivity. The combination of CEA with CA19-9, CA72-4, AFP, and novel tumor markers such as circRNA significantly enhances its diagnostic performance 94, 95. Moreover, the level of CEA is strongly linked with the prognosis of patients suffering from gastric cancer. In a study conducted by Feng et al. elevated CEA expression levels were found to be an independent risk factor for the poor outcome of early gastric cancer 93.

#### 3.1.2 Carbohydrate antigen 19-9

Carbohydrate antigen 19-9 (CA19-9) is a frequently utilized marker for the detection of colorectal cancer, with potential utility for diagnosing and forecasting outcomes in gastric cancer patients. A meta-analysis showed that CA19-9 was detected at a positive rate of 27.8% in gastric cancer, which is slightly higher than that of CEA (21.1%) 96. Moreover, CA19-9 was found to have a specificity of 74% for gastric cancer recurrence, with a sensitivity rate of 56%. Sensitivity increased up to 87% with the combination of CA19-9 and CEA 97.

#### 3.1.3 Carbohydrate antigen 72-4

The diagnostic performance of carbohydrate antigen 72-4 (CA72-4), a mucin-like glycoprotein with a high molecular weight, is slightly superior to that of other conventional serological tumor markers for identifying gastric cancer 98. A meta-analysis demonstrated that, among the single serological indicators for identifying gastric cancer, CA72-4 had the greatest diagnostic efficacy, with a sensitivity and specificity of 49% and 91%, respectively 99. In the detection of progressive gastric cancer, the positivity rate of CA72-4 is almost double that of CA19-9 (37.5% versus 17.9%) 100. When CA72-4, CA19-9 and CEA are combined, the diagnostic sensitivity increases to 67%, and the specificity is 89% 100.

#### 3.1.4 Other traditional tumor markers

Alpha fetoprotein (AFP) and carbohydrate antigen 125 (CA125) are traditional markers frequently employed to diagnose gastric cancer, with rates of positivity of 1.5% and 1.9%, respectively 93. Moreover, serum CA125 is deemed to be a beneficial prognostic biomarker in patients with unresectable, progressed or recurring gastric cancer. Carbohydrate antigen 242 (CA242) sensitivity in digestive tumors follows a descending order of hepatocellular carcinoma, gastric carcinoma, colorectal carcinoma and pancreatic carcinoma, with gastric carcinoma exhibiting a sensitivity of 11.4% 101. The expression level of carbohydrate antigen 50 (CA50), a polymeric glycoprotein with cross-immunity to CA19-9, is elevated in patients with gastric cancer, although some studies have observed comparable serum CA50 levels in patients with gastric cancer and healthy controls 102.

However, conventional biomarkers are insufficiently sensitive and specific in screening for gastric cancer, particularly in early stages. Moreover, their screening efficiency does not significantly improve when tested in combination. Therefore, conventional biomarkers are not the most effective tool for early detection and gastric cancer screening.

### 3.2 Novel biomarkers

#### 3.2.1 Polypeptides

Currently, routine laboratory tests for the diagnosis and monitoring of digestive disorders comprise four gastric function tests (PGⅠ, PGⅡ, PGⅠ/Ⅱ, and *H. pylori*-IgG antibody) and Gastrin-17 103, 104, which called biomarkers suggestive of gastric mucosal injury.

Pepsinogen (PG), the inactive pepsin precursor, can be classified into PGⅠ and PGⅡ based on its structure and immunological characteristics 105, 106. The atrophy of the gastric mucosa leads to a decline in PGⅠ/PGⅡ (i.e., PGR). It was reported to be "serological biopsy" for the gastric mucosa 107. Presently, a PGⅠ value of <70 ng/ml and a PGR value of <3 are acknowledged as the thresholds for screening gastric cancer in asymptomatic individuals 107. However, the application of PG in mass screening may lead to biased results due to its limited relevance in detecting intestinal gastric cancer 108 and the impact of *H. pylori* eradication and proton pump inhibitors on PG levels.

Gastrin-17 (G-17) is secreted by G cells located in the gastric antrum, and its serum levels are influenced by both the function of the gastric antrum and the secretion of gastric acid by parietal cells. Patients with gastric acid hypersecretion or atrophic gastric sinusitis exhibit lower levels of fasting G-17, whereas serum G-17 levels are significantly elevated in patients with gastric cancer, with its sensitivity, specificity and diagnostic accuracy in gastric cancer reaching 59.31%, 70.59% and 88.65%, respectively 109. Currently, the commonly accepted reference level is G-17 > 7 pmol/L 110. The secretion of G-17 is influenced by diet and the usage of PPIs. However, G-17 cannot be used to differentiate early gastric cancer from the progressive type. Consequently, it cannot be used independently as a serological indicator for predicting gastric cancer. Nonetheless, combining G-17 with PG and other indicators is expected to enhance the precision of gastric cancer screening 111.

Clearly, neither PG nor G-17 alone, nor a combination of both, will provide satisfactory results in gastric cancer screening. As a result, scholars worldwide have been continuously innovating based on this finding, resulting in the emergence of new combinations and permutations. For instance, Ohata et al suggested the ABC method as more appropriate for identifying high-risk groups for gastric cancer by combining serum PG and *H. pylori*-IgG antibodies 112-114. In a cohort study assessing this technique, the hazard ratios for groups B, C, and D were 1.1 (95% CI 0.4-3.4), 6.0 (2.4-14.5), and 8.2 (3.2-21.5), respectively, compared to group A 115. On the basis of these findings, *H. pylori* titers are reclassified in the ABC technique, as suggested by Kishikawa et al. and the modified ABC method proposed by Yanaoka, which further subdivides according to serum PG and *H. pylori*-IgG antibody titer levels 116-118. Furthermore, the "new ABC method", integrating G-17, has been proven beneficial for screening gastric cancer and managing high-risk groups in various cohort studies 119. It is recommended to not overlook population characteristics such as age, sex, and dietary habits, in addition to the provided laboratory indicators. Furthermore, the joint method—a predictive model combining both the ABC method and population characteristics—has shown promising results with an area under the curve of 0.76 when calibrated 120, 121.

MG7-Ag is an antigen specific to gastric cancer cells whose expression increases progressively in normal gastric mucosa, intestinalization, neoplasia and gastric cancer tissues 122. It is mainly expressed in gastric cancer cells and at low levels in other digestive tract tumors, such as colorectal cancer and esophageal cancer. We believe that it can be used not only as a relevant diagnostic indicator of gastric cancer but also as an early warning indicator of gastric cancer occurring in precancerous lesions. Over a decade ago, Zhang and colleagues demonstrated that the positive rate of MG7-Ag in patients with gastric cancer was 77.5% 123. Furthermore, various scholars have suggested that the combined detection of MG7-Ag and COX-2 is beneficial for predicting both gastric cancer and precancerous lesions 124. More recently, Wu and colleagues have also explored this topic. Additionally, a multimolecule predictive model for gastric cancer has been developed and verified that incorporates MG7-Ag 125. This indicates that MG7-Ag could serve as a serological biomarker for screening high-risk populations for gastric cancer.

The CagA protein, encoded by the *H. pylori* CagA gene, facilitates bacterial entry into the cytoplasm of gastric epithelial cells upon attachment. The presence of the CagA gene in *H. pylori* has a notable oncogenic impact and allows the bacteria to evade the host's immune system through immune escape 126. An epidemiological study of *H. pylori* carrying the CagA gene has confirmed that individuals with seropositive *H. pylori* infection and anti-CagA antibodies are at a higher risk of developing gastric cancer than those infected with negative *H. pylori* 127. Thus, the combined use of this indicator holds promise for timely intervention before patients develop cancerous or precancerous lesions.

Anti-parietal cell antibodies (APCAs), originally discovered by Taylor et al. in the serum of pernicious anemia patients, are organ-specific and do not react with any organs other than the stomach 128. These antibodies are generally regarded as biomarkers of autoimmune gastritis (AIG) and risk factors associated with atrophic gastritis, which triggers the subsequent cancerous process by inducing mucosal atrophy 128.

Hepatocyte growth factors (HGFs) exhibit significantly higher levels in patients diagnosed with gastric cancer than in the normal population. Statistics show that the rate of HGF expression in the serum of early gastric cancer patients is higher than that of CEA and CA19-9 129. Additionally, there is a correlation between the elevated level of serum HGFs in patients with early gastric cancer and lymph node metastasis, which could have the potential to become a biomarker for gastric cancer screening 129.

Tumor-associated antigens (TAAs) are proteins that emerge early in tumorigenesis. It has been reported that their autoantibody, IgG, can be detected in the bloodstream approximately 5 years prior to the clinical manifestation of the tumor. This suggests that TAAs are crucial in predicting early tumors. They have also been identified in all types of tumors that have been analyzed to date, exhibiting high antigenic specificity and stability in serum 130, 131. TAAs are qualitative biomarkers, including proteins such as p53, p62, c-Myc, PTEN, and heat shock protein 70. In recent years, several studies have been published regarding the use of TAAs to establish a predictive diagnostic model for gastric cancer. Notably, Zayakin et al. discovered 45 autoantibodies, Zhou et al. reported 7 autoantibodies against TAAs, and Wang et al. proposed 9 autoantibodies against TAAs. These autoantibodies have demonstrated the ability to differentiate between patients with gastric cancer and healthy individuals 132-134. The adaptive immune system offers significant benefits in detecting cancer in its early stages. However, additional research is necessary to expand our understanding of this promising area.

SNCG is a soluble neuroprotein that is 127 amino acids in length and belongs to the synaptic nucleoprotein gene family. It undergoes heightened expression in gastric adenocarcinomas and is related to the depth of tumor infiltration and lymph node metastasis. It was discovered that serum-derived SNCG had outstanding diagnostic value when distinguishing between patients with gastric cancer and healthy individuals. Its sensitivity, specificity, PPV, and NPV were 95.40%, 86.36%, 93.26%, and 90.48%, respectively 135.

In addition to the above, an increasing number of protein-based markers, including trefoil factor family peptides (TFFs), particularly TFF3, have been linked to gastric cancer. TFF3 demonstrates an 80.0%-80.4% sensitivity rate for detecting gastric cancer, which is significantly higher than that of PG (33.3%-39.5%) 136. The sensitivity of serum insulin-like growth factor binding protein 7 (IGFBP-7) in detecting gastric cancer was shown to be 36.7%, with a specificity of 90.0%, and the sensitivity for early gastric cancer detection was 33.3% 137. Other biomarkers, such as cytoplasmic thymidine kinase 1 (TK1), M2-pyruvate kinase, cytosolic cyclin RegIV, the inflammatory signaling molecules olfactoryin 4 and vascular adhesion protein 1 (vap-1), and serum gastric growth promoter (ghrelin), were also investigated 136, 138, 139. They have shown promise as early diagnostic indicators for gastric cancer; however, their clinical significance requires further extensive studies to be confirmed.

#### 3.2.2 DNA

Circulating free DNA (cfDNA) is genetic material originating from both normal and cancerous cells and is detectable via blood tests. ctDNA can be traced back to primary tumors, metastases or circulating tumor cells (CTCs) 140. There is evidence indicating that ctDNA can be detected in the plasma of patients with malignant tumors in the early stages of the disease and exhibits the same biological characteristics as tissue tumors 141. This suggests that cfDNA from tumor patients is mainly derived from ctDNA, while cfDNA from healthy individuals is mainly derived from blood cells 142.

Multiple studies have demonstrated that circulating ctDNA levels in cancer patients are generally higher than those in healthy individuals, indicating potential diagnostic significance. Qian et al conducted a study analyzing the levels of cfDNA in the serum of 124 individuals diagnosed with gastric cancer, 64 diagnosed with benign gastric disease (BGD), and 92 healthy controls 143. The findings revealed that the levels of cfDNA in GC patients were significantly higher than those in BGD patients and healthy controls 143. Park et al discovered that the mean plasma cfDNA concentration in a group of 54 GC patients and 59 age-matched healthy controls was approximately 2.4 times higher in the GC group than in the control group 144. In addition, another study showed that cfDNA levels significantly decreased 24 hours after surgery when compared to preoperative values 145.

However, studies indicate that ctDNA testing possesses specific advantages for gastric cancer diagnosis, with undetectable ctDNA performing better than conventional protein biomarkers such as CEA, CA125, and CA724 in terms of sensitivity. Furthermore, it has been observed that increased levels of cfDNA are present in individuals with cardiovascular, infectious, and inflammatory conditions and in healthy individuals following physical activity, such as running a marathon. This suggests that the phenomenon is not solely restricted to cancer patients 146. Currently, the most extensively researched matter in cfDNA studies concerns the role of ctDNA in cancer therapy, with further refinement needed for its application in gastric cancer diagnosis, particularly in early gastric cancer screening.

DNA methylation is the initial epigenetic mark, which is crucial in tumorigenesis, as it provides a steady mechanism of gene suppression and regulates gene expression and chromatin structure. It is linked with histone modifications and other chromatin-related proteins 140. Furthermore, DNA methylation is the most extensively researched epigenetic modification and is the most precisely defined epigenetic feature of gastric cancer. Studies have demonstrated that the identification of hypermethylated genes in serum DNA samples of patients could indicate early detection and prediction of gastric cancer. Examples of these genes include promoter methylation of the p16 gene, RASSF1A methylation, Runt-associated transcription factor 3 (RUNX3) methylation and Reprimo methylation 147-149.

Epigenetic alterations are typically considered early events in gastric carcinogenesis, which may precede pretumor or early tumor stages, such as DNA hypomethylation and CpG island hypermethylation. They may also serve as indicators or biomarkers for screening patients at an increased risk of developing GC. Ren et al. conducted a genome-scale DNA methylation analysis of gastric cancer patients (*n*=89) and control participants (*n*=82) as well as 28 pairs of GC and adjacent noncancerous tissues, using MCTA-Seq 147. The study evaluated the efficacy of DNA methylation in detecting gastrointestinal cancers (GCs) and distinguishing between colorectal cancer (CRC) and hepatocellular carcinoma (HCC). In total, the research identified 153 cfDNA methylation biomarkers, such as DOCK10, CABIN1, and KCNQ5 147. These biomarkers displayed sensitivities of 44%, 59%, 78%, and 100% for stage I, II, III, and IV tumors, respectively, with specificities of 92% 147. Furthermore, the study found that CpG island methylation phenotype (CIMP) tumors and non-CIMP tumors could be accurately distinguished and detected. The results demonstrate that MCTA-Seq can accurately differentiate between early-stage GC, CRC, and HCC in the bloodstream using a high-specificity algorithm 147. These findings suggest that the methylation of cfDNA holds promise as a noninvasive blood-based diagnostic tool for GC.

However, there is a lack of agreement on the standard process used for experimental procedures to detect cfDNA. Furthermore, there is considerable overlap in the amount of DNA obtained from the plasma of both healthy individuals and cancer patients when comparing plasma and serum samples. This phenomenon is particularly evident in serum samples, which complicates the distinction between healthy individuals and cancer patients 150. Although there are constraints, utilizing cfDNA levels alongside other biomarkers has the potential to enhance the effectiveness of screening.

Microsatellite instability (MSI) is a molecular phenotype resulting from defects in the DNA mismatch repair (MMR) mechanism. MSI is manifested by abnormal lengths (increase or decrease) of microsatellite repeat sequences 151. In 2014, The Cancer Genome Atlas (TCGA) study classified gastric adenocarcinoma into the following four molecular phenotypes based on comprehensive molecular analysis: EBV-positive; high microsatellite instability (MSI-H); genomically stable (GS); and chromosomal instability (CIN), with MSI-H accounting for 22% of all cases. MSI-H is the second most common type after CIN, which accounts for the majority of cases 152. Recent research conducted both domestically and internationally demonstrates that MSI-H tumors carry a positive prognosis, and it has also been hypothesized that MSI status could be indicative of a tumor's response to chemotherapy during stage II/III gastric cancer 153, 154.

Nonetheless, there is currently no compelling evidence that MSI status plays a significant role in gastric cancer detection. A total of 1,156 tumors were analyzed in a retrospective study, of which 85 (7.4%) were MSI-H tumors. Among submucosal gastric cancers, lymphovascular infiltration (LVI) was found to occur more frequently in MSI-H tumors than in MSS tumors (38.9% vs. 25.0%) 155. However, both types of tumors had similar prognoses (log-rank test; hazard ratio for MSI-H adjusted for age, sex, pT stage, and the number of metastatic LNs was 0.932 with a 95% confidence interval of 0.423-2.054 and a p value of 0.861) 155. Nonetheless, the regular incidence of lymphovascular infiltration (LVI) in MSI-H gastric cancer, among other factors, may assist in directing patients toward prompt and suitable treatments, including endoscopic procedures or restricted surgical removal of lymph nodes 155.

#### 3.2.3 Noncoding RNA

MicroRNAs (miRNAs) are noncoding RNAs of approximately 20-24 nucleotides in length that can control the expression of target genes through interference or inhibition of transcription and participate in most biological events, such as tumorigenesis 156. Over recent decades, numerous studies have suggested that miRNAs play a role in the development of gastric cancer. They are a type of noncoding RNA that can be found in patients' blood, gastric fluid and other body fluids. Furthermore, miRNAs present in peripheral blood are considered promising novel biomarkers for gastric cancer screening 157.

Li and colleagues discovered a downregulation of miR-381 expression in the blood of patients with gastric cancer 158. Additionally, they identified other conventional biomarkers in the patients' blood. The AUC of miR-381 was found to be 0.931, with AUCs of CA199, CA724, and CEA measuring 0.761, 0.843, and 0.788, respectively; thus, it was demonstrated that the diagnostic performance of miRNA-381 was significantly superior to that of the other three biomarkers. Similar findings were observed in other studies concerning miR-17, miR-25, miR-196a/b, and so on 159-161. Our group also found some miRNAs closely related to gastric cancer, such as miR-195, miR-378 and miR-421, which have potential to become biomarkers for gastric cancer screening 162, 163.

Gastric cancer is a complex illness, and the use of single miRNAs for diagnosis appears restricted. Therefore, several academics have proposed a gastric cancer screening predictive model founded on multiple miRNAs to address this challenge. Izumi et al's study, divided into biomarker discovery, tissue validation, retrospective serum validation, and multicenter prospective serum performance evaluation phases, presented a prediction model that employs 3 miRNAs (miR-18a, miR-181b, and miR-335) to enhance the early detection rates of gastric cancer among high-risk populations (AUC, 0.87; 95% CI, 0.83-0.92) 164. Abe et al. proposed four miRNAs (miR-4257, miR-6785-5p, miR-187-5p, and miR-5739), which achieved an AUC of 0.996 165. Similarly, Zhu et al proposed five miRNAs (miR-16, miR-25, miR92a, miR-451, and miR-4865p) achieving an AUC of 0.989 166. Huang et al. proposed six miRNAs (miR10b-5p, miR132-3p, miR185-5p, miR195-5p, miR-20a3p, miR296-5p), obtaining an AUC of 0.764 167.

To enhance the screening effectiveness of miRNA-based gastric cancer prediction models, combining multiple miRNAs and exploring other combinations, such as traditional biomarkers or novel biomarker lncRNAs, circRNAs, etc., should be considered. In conclusion, further research and exploration are needed to establish the application of miRNAs as novel biomarkers in clinical and large-scale gastric cancer screening, but their potential is promising.

Long noncoding RNA (lncRNA) refers to a class of RNA molecules with a transcript length exceeding 200 nucleotides (nt) that do not have protein-coding functions 168. LncRNAs, previously regarded as "transcriptional noise", have been demonstrated to participate in gene imprinting, chromatin modification, transcription, activation, interference, cell cycle regulation, splicing, translation and other cellular processes, performing crucial functions 169, 170. Numerous domestic and international studies have identified numerous lncRNAs that are closely linked to gastric cancer. RMRP, GClnc1, and HCP5, among others, have been found to regulate gene expression at various levels and contribute to tumorigenesis and development. They are also closely connected to tumor invasion, metastasis, and patient prognosis 171, 172.

The RNA component of the mitochondrial RNA processing endoribonuclease (RMRP), which is situated on human chromosome 9p13.3, is a lncRNA molecule that extends to a complete length of 277 nucleotides. It has various biological functions. Our group found that lncRNA RMRP plays a significant role in the development and carcinogenesis of gastric cancer 173. We examined its molecular mechanism in gastric tumorigenesis and then demonstrated that using RMRP as a serological biomarker for screening gastric cancer gave sensitivity and specificity scores of 59.1% and 67.8%, respectively 173. Plasma RMRP levels were lower in healthy individuals, gastric cancer patients had significantly increased plasma RMRP levels conversely. Moreover, plasma RMRP levels decreased significantly after the surgical removal of damaged tissues in the patients, indicating plasma RMRP's potential as a biomarker in gastric cancer screening and prognostic evaluation.

GClnc1 also exhibits high accuracy in detecting EGC. Through a genome-wide transcriptome analysis and differing between EGC and precancerous lesions in a multicenter validation analysis, the AUC of GClnc1 exceeded 0.87 171. The sensitivity exceeded 86%, and the specificity exceeded 76% across all three independent validation cohorts 171. Serum-derived UCA1, PCGEM1, and CUDR are potential new biomarkers for the early detection of gastric cancer, as confirmed by qRT‒PCR and other techniques 174-176.

Similar to miRNAs, combining multiple lncRNAs or using them in conjunction with other biomarker classes can enhance diagnostic accuracy for gastric cancer screening. Dong et al discovered that a screening model featuring a combination of three lncRNAs (CUDR, LSINCT-5, and PTENP1) effectively differentiated between healthy individuals and those suffering from gastric cancer (AUC=0.920, accuracy 87.1%, sensitivity 74.1%, specificity 100%) 176. This model serves as a more precise diagnostic biomarker than CA19-9 and CEA. Chen et al. conducted a study on a combination of four lncRNAs (CEBPA-AS1, INHBA-AS1, AK001058, and UCA1) and two miRNAs (PPBP and RGS18) 177. They discovered that the combination of three lncRNAs (INHBA-AS1, AK001058, and UCA1) and one miRNA (RGS18) had the best diagnostic performance, with an AUC of 0.820, sensitivity of 0.782, and specificity of 0.708 177.

The aforementioned findings indicate that lncRNAs hold potential for the timely detection of gastric cancer. However, the efficacy of these molecules as novel biomarkers necessitates additional research and refinement before optimal integration into clinical settings.

Circular RNA (circRNA) pertains to a category of endogenous, circular RNA molecules created via variable splicing of precursor RNA and tethered by 5'-terminal and 3'-terminal converse covalent bonds 178. These molecules are characterized by their high abundance, stability, conservation, and specificity to certain tissues 178. Notably, circRNA exhibits these traits without involving encoding proteins and may perform multiple functions in gene expression regulation. These molecules have the potential to regulate gene expression through transcriptional regulation, acting as sponges for miRNA molecules, protein translation, and interacting with RNA-binding proteins (RBPs) to govern downstream molecular pathways 178. Furthermore, they play an essential role in the development of gastric cancer 179-183.

CircRNAs are abundant in both tissues and bodily fluids, such as blood, gastric fluid, and saliva 184. In gastric cancer, circRNAs display differential expression. Many scientists tried to figure out the diagnostic value of plasma-based circRNAs in comprehensive early gastric cancer screening. In their cohort study conducted in Japan, Roy et al created and tested predictive models for potential circRNAs in both matched GC and adjacent normal mucosal tissue specimens 185. They then employed a diagnostic risk prediction model for GC utilizing eight circRNAs in serum samples from relevant patients, subsequently proving that this strategy could effectively differentiate them from nondiseased controls, with an AUC value of 0.87, sensitivity of 78.3% and specificity of 78.3% 185. Even individuals with early-stage gastric cancer can be accurately identified irrespective of their tumor histology. This offers compelling and encouraging evidence for the clinical use of circRNA as an innovative biomarker in the early diagnosis of gastric cancer patients.

Hsa_circ_0001020 is a 1631-nucleotide circRNA. According to our group's series of studies, the plasma levels of hsa_circ_0001020 increased among people with gastric cancer 95. Nevertheless, its level significantly diminished two weeks after surgery 95. When used as a single biomarker for gastric cancer screening, plasma hsa_circ_0001020 showed a higher AUC value (0.738) than CEA (0.560) and CA72-4 (0.670) 95. It also exhibited a better sensitivity of 46.55% in contrast to conventional biomarkers such as CEA (21.1%), CA19-9 (27.8%) and CA72-4 (30.0%) 95. Combined with CEA and CA19-9, the AUC, sensitivity and specificity values were 0.852, 68.5% and 89.1%, respectively 95. Moreover, our group found that hsa_circ_0000419 showed sensitivity and specificity values of 0.682 and 0.884 when used for screening gastric cancer 181, while hsa_circ_0086720 demonstrated up to 67.4% sensitivity and 87.2% specificity for early gastric cancer 179. There is strong evidence to suggest that both of them are potential novel screening biomarkers for gastric cancer.

Currently, numerous circRNAs are undergoing tissue-based discovery to ascertain their potential as blood (serum or plasma) biomarkers. Additionally, some circRNAs remain unvalidated in independent cohorts with large clinical samples. circRNAs offer the benefits of easy detection, good diagnostic performance, and promising early screening for gastric cancer.

#### 3.2.4 Other serological biomarkers

Exosomes are bilayer lipid vesicles enclosed in a membrane that cells secrete. They contain an array of substances, such as nucleic acids, proteins, and enzymes, allowing them to transport various molecular signals that range from RNAs to proteins between cells 186. Exosomal RNAs encompass lncRNAs, circRNAs, miRNAs, and others 186.

Serum exosomal miR-1246 expression can be used to differentiate patients with TNM stage I gastrointestinal cancer (GC) from both healthy controls (HCs) and patients with benign diseases (BDs) with areas under the curve (AUCs) of 0.843 and 0.811, respectively 187. The RNA Pol II transcript, lncRNA-GCI, has also demonstrated significant potential as an early detection biomarker for GC and holds promise as a disease progression biomarker 188. Another study discovered that patients with gastric cancer exhibit significantly elevated levels of three serum EV-derived circRNAs (Chr10q11, Chr1p11, and Chr7q11) compared to those of healthy controls 189. Furthermore, when used in conjunction with CEA, the combination resulted in an area under the curve (AUC) of 0.866 (95% CI: 0.803-0.915) with a corresponding sensitivity and a specificity of 80.4% and 81.8%, respectively 189.

Exosomes, as bilayer lipid vesicles, offer protection to their transported RNA from degradation by nucleases and maintain stability under varying temperature and pH conditions. Consequently, exosomes secreted by tumor cells can indicate the presence of cancer and its developmental changes with more precision. As a result, they hold vital clinical potential as biomarkers for gastric cancer screening.

Circulating tumor cells (CTCs) originate from solid tumors, separate from the primary tumor, and enter body fluids through the vascular system. Previous research has indicated that they are a significant factor in cancer recurrence and metastasis 190. CTCs were identified a century ago, but only in recent years has their potential as a biomarker for the early detection of gastric cancer been demonstrated, particularly through the analysis of autoantibodies. This use in early diagnosis is crucial. Kang et al carried out a prospective study of 116 gastric cancer patients and 31 healthy volunteers, confirming that 99 of the patients (97% of the study population) had positive CTC and autoantibody expression levels 191. Only 1% of the 102 individuals with CTC levels of ≥2 CTCs/7.5 mL of blood were found to have gastric cancer 191. Furthermore, the sensitivity and specificity of CTCs in distinguishing patients with gastric cancer from healthy control subjects were 85.3% and 90.3%, respectively 191. Furthermore, a recent study discovered that CTCs were present in 47.89% (34/71) of patients with EGC/gastric precancerous lesions and in 4.76% (1/21) of patients with basal gland polyps 192. This provides additional evidence that assessing peripheral blood CTCs is a valuable method for assisting in detecting EGC and precancerous lesions 192.

The neutrophil-lymphocyte ratio (NLR) and platelet-lymphocyte ratio (PLR) are widely recognized to be effective biomarkers of systemic inflammation. In a recent retrospective study, Fang and colleagues examined 2,606 patients newly diagnosed with gastric cancer in the past three years as well as 3,219 healthy controls during the same period 193. A comprehensive analysis of peripheral blood samples was undertaken for NLR, PLR, CEA and CA19-9, and the study provides valuable insight into these inflammatory biomarkers for cancer patients 193. The diagnostic significance of NLR and PLR was found to be superior to that of CEA and CA19-9 for diagnosing gastric cancer 193. Moreover, upon grouping by sex, the diagnostic significance of NLR and PLR for GC was found to be greater in male patients 193.

## Perspectives

Gastric cancer is a progressive and multistage disease. The prognosis of advanced gastric cancer is poor and consumes a large amount of limited medical resources, whereas early gastric cancer can be endoscopically resected. Therefore, how to efficiently and economically screen high-risk groups for gastric cancer as well as provide accurate assessment and dynamic follow-up is the key to achieving early diagnosis and treatment of gastric cancer. With the emergence of more new gastric cancer biomarkers and the technological improvement of imaging and endoscopic methods, existing clinical strategies for cancer screening have been complemented, and new options have been provided. However, the optimization of cancer screening protocols for different regions and populations still needs to be tested and explored in practice and clinical research.

Medical imaging is a field that has developed over the years and has achieved a certain level of maturity. However, it often falls short in providing adequate visualization of early lesions. The advent of molecular imaging technology is expected to overcome this challenge. Unlike traditional imaging methods, molecular imaging technology could identify cellular and molecular level abnormalities during a disease and detect such changes before any anatomical alterations occur. This feature is advantageous in detecting gastric cancer at an early stage. For example, the application of LGR5-targeting peptide probe enables the fluorescence rate of gastric cancer cells is 2 to 10 times higher than that of control cells 194.

Endoscopy and pathological biopsy remain the essential and standard methods for diagnosing gastric cancer. However, their invasiveness and dependence on equipment and technique pose certain limitations. Currently, the diagnosis of gastric cancer follows a fixed procedure. First, high-risk patients undergo an initial screening with white light endoscopy. Based on this, comprehensive consideration is given to the size, location and depth of the lesion, followed by the selection of appropriate endoscopic techniques for further confirmation of the diagnosis. The rise in painless endoscopy has resulted in a greater acceptance of the procedure. Additionally, computer-aided diagnostic technology has addressed some limitations of traditional endoscopy.

Although biomarkers offer a noninvasive, simple and affordable option for mass screening, the diagnostic capabilities of established biomarkers are unsatisfactory. There is a scarcity of biomarkers exhibiting high sensitivity and specificity, such as PSA for prostate cancer and AFP for liver cancer. Various potential new serological biomarkers for gastric cancer are still in their nascent basic research phase. To establish their sensitivity, specificity, and accuracy in gastric cancer screening, more large-sample clinical cohort studies are necessary in the future, along with in-depth research on the specific molecular mechanisms and pathways of the role of these markers in the development of gastric cancer. Recent years, tRNA-derived small RNA (tsRNA) have been a new hot topic in the screening of gastric cancer. Our group has conducted extensive research and identified several tsRNAs, such as tRF-19-3L7L73JD, tRF-33-P4R8YP9LON4VDP, and tRF-5026a, as potential biomarkers for early gastric cancer 194-197. Undoubtedly, serological biomarkers that demonstrate superior diagnostic performance will aid in the early detection of gastric cancer.

Nowadays, there has been significant interest in tumor-associated microbiota and endogenous metabolic small molecules. Some of these microbiota and molecules may serve as markers for gastric cancer screening. However, few reports have been published on their use in early gastric cancer screening. Regarding the current state of research, the diagnostic performance of individual microbiota or metabolites is not optimal. Often, a combination of multiple markers is necessary to improve their sensitivity and specificity. Additionally, these substances, particularly metabolites, are unstable in plasma and are susceptible to various factors. In conclusion, the use of these markers for gastric cancer screening presents several challenges that require resolution and further research and clinical confirmation.

All approaches have their strengths and limitations, and a combination of methods is often used in clinical practice to test and fill the gaps and maximize the benefits. For example, a clinical study shows that a gastric cancer screening system with high efficacy can be established by using a combination of the PG test and barium DR 199. Furthermore, Cost-effective and straightforward serological screening methods could be utilized to initiate the screening of large cohorts. When coupled with various risk factors, such as sex, age, and living habits, gastric cancer risk stratification can be carried out. Consequently, high-risk groups can undergo endoscopy and pathological biopsy followed by appropriate treatment and follow-up. This process aims to enhance early gastric cancer diagnosis rates and patient survival rates.

In summary, the current major screening methods for early gastric cancer possess both strengths and limitations, and none have achieved ideal perfection. However, with the ongoing progress of technology and biological multiomics research, along with the merging and restructuring of novel and existing methods, it is hoped that the global rate of early gastric cancer detection will greatly improve and confer a decrease in the mortality rate worldwide.

## References

[1] Sung H, Ferlay J, Siegel RL, Laversanne M, Soerjomataram I, Jemal A, Bray F (2021). Global Cancer Statistics 2020: GLOBOCAN Estimates of Incidence and Mortality Worldwide for 36 Cancers in 185 Countries. CA Cancer J Clin.

[2] Ferlay J, Colombet M, Soerjomataram I, Parkin DM, Piñeros M, Znaor A, Bray F (2021). Cancer statistics for the year 2020: An overview. Int J Cancer.

[3] Wang FH, Zhang XT, Li YF, Tang L, Qu XJ, Ying JE, Zhang J, Sun LY, Lin RB, Qiu H (2021). The Chinese Society of Clinical Oncology (CSCO): Clinical guidelines for the diagnosis and treatment of gastric cancer, 2021. Cancer Commun (Lond).

[4] Chiarello MM, Fico V, Pepe G, Tropeano G, Adams NJ, Altieri G, Brisinda G (2022). Early gastric cancer: A challenge in Western countries. World J Gastroenterol.

[5] Xu H, Li W (2022). Early detection of gastric cancer in China: progress and opportunities. Cancer Biol Med.

[6] Song Z, Wu Y, Yang J, Yang D, Fang X (2017). Progress in the treatment of advanced gastric cancer. Tumour Biol.

[7] Isobe Y, Nashimoto A, Akazawa K, Oda I, Hayashi K, Miyashiro I, Katai H, Tsujitani S, Kodera Y, Seto Y, Kaminishi M (2011). Gastric cancer treatment in Japan: 2008 annual report of the JGCA nationwide registry. Gastric Cancer.

[8] Nashimoto A, Akazawa K, Isobe Y, Miyashiro I, Katai H, Kodera Y, Tsujitani S, Seto Y, Furukawa H, Oda I (2013). Gastric cancer treated in 2002 in Japan: 2009 annual report of the JGCA nationwide registry. Gastric Cancer.

[9] Kim H, Hwang Y, Sung H, Jang J, Ahn C, Kim SG, Yoo KY, Park SK (2018). Effectiveness of Gastric Cancer Screening on Gastric Cancer Incidence and Mortality in a Community-Based Prospective Cohort. Cancer Res Treat.

[10] Teh JL, Shabbir A, Yuen S, So JB (2020). Recent advances in diagnostic upper endoscopy. World J Gastroenterol.

[11] Teufel A, Quante M, Kandulski A, Hirth M, Zhan T, Eckardt M, Thieme R, Kusnik A, Yesmembetov K, Wiest I (2021). [Prevention of gastrointestinal cancer]. Z Gastroenterol.

[12] Young JW, Ginthner TP, Keramati B (1985). The competitive barium meal. Clin Radiol.

[13] Hisamichi S (1989). Screening for gastric cancer. World J Surg.

[14] Dooley CP, Larson AW, Stace NH, Renner IG, Valenzuela JE, Eliasoph J, Colletti PM, Halls JM, Weiner JM (1984). Double-contrast barium meal and upper gastrointestinal endoscopy. A comparative study. Ann Intern Med.

[15] James WB, McCreath G, Sutherland GR, McDonald M (1976). Double contrast barium meal examination - a comparison of techniques for introducing gas. Clin Radiol.

[16] Yamamichi N, Shimamoto T, Hirano C, Takahashi Y, Minatsuki C, Takeuchi C, Takahashi M, Sakaguchi Y, Tsuji Y, Niimi K (2021). Clinicopathological features and prognosis of developed gastric cancer based on the diagnosis of mucosal atrophy and enlarged folds of stomach by double-contrast upper gastrointestinal barium X-ray radiography. Clin J Gastroenterol.

[17] Ballantyne KC, Morris DL, Jones JA, Gregson RH, Hardcastle JD (1987). Accuracy of identification of early gastric cancer. Br J Surg.

[18] Yoshida Y, Yamaguchi Y, Tebayashi A, Arisue T, Tamura K, Ichikawa H (1983). Precision in the first stage of the gastric carcinoma mass survey. Int J Cancer.

[19] Kubota H, Kotoh T, Masunaga R, Dhar DK, Shibakita M, Tachibana M, Kohno H, Nagasue N (2000). Impact of screening survey of gastric cancer on clinicopathological features and survival: retrospective study at a single institution. Surgery.

[20] Xu Q, Sun Z, Li X, Ye C, Zhou C, Zhang L, Lu G (2021). Advanced gastric cancer: CT radiomics prediction and early detection of downstaging with neoadjuvant chemotherapy. Eur Radiol.

[21] Zhen Y, Xie Q, Liu L (2022). Diagnostic Value of Spiral CT and Magnetic Resonance Imaging Scanning in Gastric Cancer and Precancerous Lesions. Scanning.

[22] Morrin MM, Farrell RJ, Kruskal JB, Reynolds K, McGee JB, Raptopoulos V (2000). Utility of intravenously administered contrast material at CT colonography. Radiology.

[23] Zeng Q, Feng Z, Zhu Y, Zhang Y, Shu X, Wu A, Luo L, Cao Y, Xiong J, Li H (2022). Deep learning model for diagnosing early gastric cancer using preoperative computed tomography images. Front Oncol.

[24] Shimizu K, Ito K, Matsunaga N, Shimizu A, Kawakami Y (2005). Diagnosis of gastric cancer with MDCT using the water-filling method and multiplanar reconstruction: CT-histologic correlation. AJR Am J Roentgenol.

[25] Kawanami S, Komori M, Tsurumaru D, Matsuura S, Nishie A, Honda H (2011). Description of early gastric cancer with wall-carving technique on multidetector computed tomography. Jpn J Radiol.

[26] Lee DH (2000). Two-dimensional and three-dimensional imaging of gastric tumors using spiral CT. Abdom Imaging.

[27] M DIG, Carbonetti F, Bonome P, Grossi A, Mazzuca F, Masoni L (2020). Hydro-MDCT for Gastric Adenocarcinoma Staging. A Comparative Study With Surgical and Histopathological Findings for Selecting Patients for Echo-endoscopy. Anticancer Res.

[28] Joo I, Lee JM, Han JK, Yang HK, Lee HJ, Choi BI (2015). Dynamic contrast-enhanced MRI of gastric cancer: Correlation of the perfusion parameters with pathological prognostic factors. J Magn Reson Imaging.

[29] Frydrychowicz A, Lubner MG, Brown JJ, Merkle EM, Nagle SK, Rofsky NM, Reeder SB (2012). Hepatobiliary MR imaging with gadolinium-based contrast agents. J Magn Reson Imaging.

[30] Heyn C, Sue-Chue-Lam D, Jhaveri K, Haider MA (2012). MRI of the pancreas: problem solving tool. J Magn Reson Imaging.

[31] Zhang XP, Tang L, Sun YS, Li ZY, Ji JF, Li XT, Liu YQ, Wu Q (2012). Sandwich sign of Borrmann type 4 gastric cancer on diffusion-weighted magnetic resonance imaging. Eur J Radiol.

[32] Tang L, Sun YS, Li ZY, Cao K, Zhang XY, Li XT, Ji JF (2016). Diffusion-weighted magnetic resonance imaging in the depiction of gastric cancer: initial experience. Abdom Radiol (NY).

[33] Jang KM, Kim SH, Lee SJ, Lee MW, Choi D, Kim KM (2014). Upper abdominal gadoxetic acid-enhanced and diffusion-weighted MRI for the detection of gastric cancer: Comparison with two-dimensional multidetector row CT. Clin Radiol.

[34] Moreno CC, Sullivan PS, Kalb BT, Tipton RG, Hanley KZ, Kitajima HD, Dixon WT, Votaw JR, Oshinski JN, Mittal PK (2015). Magnetic resonance imaging of rectal cancer: staging and restaging evaluation. Abdom Imaging.

[35] Jha RC, Zanello PA, Ascher SM, Rajan S (2014). Diffusion-weighted imaging (DWI) of adenomyosis and fibroids of the uterus. Abdom Imaging.

[36] Zheng XZ, Zhang LJ, Wu XP, Lu WM, Wu J, Tan XY (2017). Oral Contrast-Enhanced Gastric Ultrasonography in the Assessment of Gastric Lesions: A Large-Scale Multicenter Study. J Ultrasound Med.

[37] Liu Z, Guo J, Wang S, Zhao Y, Li J, Ren W, Tang S, Xie L, Huang Y, Sun S, Huang L (2015). Evaluation of transabdominal ultrasound after oral administration of an echoic cellulose-based gastric ultrasound contrast agent for gastric cancer. BMC Cancer.

[38] He X, Sun J, Huang X, Zeng C, Ge Y, Zhang J, Wu J (2017). Comparison of Oral Contrast-Enhanced Transabdominal Ultrasound Imaging With Transverse Contrast-Enhanced Computed Tomography in Preoperative Tumor Staging of Advanced Gastric Carcinoma. J Ultrasound Med.

[39] Fairweather M, Jajoo K, Sainani N, Bertagnolli MM, Wang J (2015). Accuracy of EUS and CT imaging in preoperative gastric cancer staging. J Surg Oncol.

[40] Zhang Y, Zhang J, Yang L, Huang S (2021). A meta-analysis of the utility of transabdominal ultrasound for evaluation of gastric cancer. Medicine (Baltimore).

[41] Liu Z, Guo J, Li J, Wang S, Tang S, Xie L, Huang Y, Lu W, Ren W, Sun S, Huang L (2016). Gastric Lesions: Demonstrated by Transabdominal Ultrasound After Oral Administration of an Echoic Cellulose-Based Gastric Ultrasound Contrast Agent. Ultraschall Med.

[42] Lim JH (2000). Ultrasound examination of gastrointestinal tract diseases. J Korean Med Sci.

[43] Panteris V, Nikolopoulou S, Lountou A, Triantafillidis JK (2014). Diagnostic capabilities of high-definition white light endoscopy for the diagnosis of gastric intestinal metaplasia and correlation with histologic and clinical data. Eur J Gastroenterol Hepatol.

[44] Pimenta-Melo AR, Monteiro-Soares M, Libânio D, Dinis-Ribeiro M (2016). Missing rate for gastric cancer during upper gastrointestinal endoscopy: a systematic review and meta-analysis. Eur J Gastroenterol Hepatol.

[45] Singh R, Chiam KH, Leiria F, Pu L, Choi KC, Militz M (2020). Chromoendoscopy: role in modern endoscopic imaging. Transl Gastroenterol Hepatol.

[46] Areia M, Amaro P, Dinis-Ribeiro M, Cipriano MA, Marinho C, Costa-Pereira A, Lopes C, Moreira-Dias L, Romãozinho JM, Gouveia H (2008). External validation of a classification for methylene blue magnification chromoendoscopy in premalignant gastric lesions. Gastrointest Endosc.

[47] Ogawa R, Nishikawa J, Hideura E, Goto A, Koto Y, Ito S, Unno M, Yamaoka Y, Kawasato R, Hashimoto S (2019). Objective Assessment of the Utility of Chromoendoscopy with a Support Vector Machine. J Gastrointest Cancer.

[48] Sakai Y, Eto R, Kasanuki J, Kondo F, Kato K, Arai M, Suzuki T, Kobayashi M, Matsumura T, Bekku D (2008). Chromoendoscopy with indigo carmine dye added to acetic acid in the diagnosis of gastric neoplasia: a prospective comparative study. Gastrointest Endosc.

[49] Lee BE, Kim GH, Park DY, Kim DH, Jeon TY, Park SB, You HS, Ryu DY, Kim DU, Song GA (2010). Acetic acid-indigo carmine chromoendoscopy for delineating early gastric cancers: its usefulness according to histological type. BMC Gastroenterol.

[50] Davila RE (2009). Chromoendoscopy. Gastrointest Endosc Clin N Am.

[51] Kurumi H, Nonaka K, Ikebuchi Y, Yoshida A, Kawaguchi K, Yashima K, Isomoto H (2021). Fundamentals, Diagnostic Capabilities and Perspective of Narrow Band Imaging for Early Gastric Cancer. J Clin Med.

[52] Yoshida N, Doyama H, Yano T, Horimatsu T, Uedo N, Yamamoto Y, Kakushima N, Kanzaki H, Hori S, Yao K (2021). Early gastric cancer detection in high-risk patients: a multicentre randomised controlled trial on the effect of second-generation narrow band imaging. Gut.

[53] Yao K, Anagnostopoulos GK, Ragunath K (2009). Magnifying endoscopy for diagnosing and delineating early gastric cancer. Endoscopy.

[54] Ezoe Y, Muto M, Uedo N, Doyama H, Yao K, Oda I, Kaneko K, Kawahara Y, Yokoi C, Sugiura Y (2011). Magnifying narrowband imaging is more accurate than conventional white-light imaging in diagnosis of gastric mucosal cancer. Gastroenterology.

[55] Matsumoto K, Ueyama H, Yao T, Abe D, Oki S, Suzuki N, Ikeda A, Yatagai N, Akazawa Y, Komori H (2020). Diagnostic limitations of magnifying endoscopy with narrow-band imaging in early gastric cancer. Endosc Int Open.

[56] Nagahama T, Yao K, Maki S, Yasaka M, Takaki Y, Matsui T, Tanabe H, Iwashita A, Ota A (2011). Usefulness of magnifying endoscopy with narrow-band imaging for determining the horizontal extent of early gastric cancer when there is an unclear margin by chromoendoscopy (with video). Gastrointest Endosc.

[57] Nagahama T, Yao K, Uedo N, Doyama H, Ueo T, Uchita K, Ishikawa H, Kanesaka T, Takeda Y, Wada K (2018). Delineation of the extent of early gastric cancer by magnifying narrow-band imaging and chromoendoscopy: a multicenter randomized controlled trial. Endoscopy.

[58] Takahashi H, Miura Y, Osawa H, Takezawa T, Ino Y, Okada M, Lefor AK, Yamamoto H (2019). Blue Laser Imaging with a Small-Caliber Endoscope Facilitates Detection of Early Gastric Cancer. Clin Endosc.

[59] Dohi O, Yagi N, Naito Y, Fukui A, Gen Y, Iwai N, Ueda T, Yoshida N, Kamada K, Uchiyama K (2019). Blue laser imaging-bright improves the real-time detection rate of early gastric cancer: a randomized controlled study. Gastrointest Endosc.

[60] Dohi O, Yagi N, Majima A, Horii Y, Kitaichi T, Onozawa Y, Suzuki K, Tomie A, Kimura-Tsuchiya R, Tsuji T (2017). Diagnostic ability of magnifying endoscopy with blue laser imaging for early gastric cancer: a prospective study. Gastric Cancer.

[61] Dohi O, Yagi N, Yoshida S, Ono S, Sanomura Y, Tanaka S, Naito Y, Kato M (2017). Magnifying Blue Laser Imaging versus Magnifying Narrow-Band Imaging for the Diagnosis of Early Gastric Cancer: A Prospective, Multicenter, Comparative Study. Digestion.

[62] Yoshifuku Y, Sanomura Y, Oka S, Kuroki K, Kurihara M, Mizumoto T, Urabe Y, Hiyama T, Tanaka S, Chayama K (2017). Clinical Usefulness of the VS Classification System Using Magnifying Endoscopy with Blue Laser Imaging for Early Gastric Cancer. Gastroenterol Res Pract.

[63] Castro R, Rodriguez M, Libânio D, Esposito G, Pita I, Patita M, Santos C, Pimentel-Nunes P, Dinis-Ribeiro M (2019). Reliability and accuracy of blue light imaging for staging of intestinal metaplasia in the stomach. Scand J Gastroenterol.

[64] Umegaki E, Misawa H, Handa O, Matsumoto H, Shiotani A (2023). Linked Color Imaging for Stomach. Diagnostics (Basel).

[65] Yashima K, Onoyama T, Kurumi H, Takeda Y, Yoshida A, Kawaguchi K, Yamaguchi N, Isomoto H (2023). Current status and future perspective of linked color imaging for gastric cancer screening: a literature review. J Gastroenterol.

[66] Fukuda H, Miura Y, Osawa H, Takezawa T, Ino Y, Okada M, Khurelbaatar T, Lefor AK, Yamamoto H (2019). Linked color imaging can enhance recognition of early gastric cancer by high color contrast to surrounding gastric intestinal metaplasia. J Gastroenterol.

[67] Shinozaki S, Osawa H, Hayashi Y, Lefor AK, Yamamoto H (2019). Linked color imaging for the detection of early gastrointestinal neoplasms. Therap Adv Gastroenterol.

[68] Yamaoka M, Imaeda H, Miyaguchi K, Ashitani K, Tsuzuki Y, Ohgo H, Soma H, Hirooka N, Nakamoto H (2021). Detection of early stage gastric cancers in screening laser endoscopy using linked color imaging for patients with atrophic gastritis. J Gastroenterol Hepatol.

[69] Yasuda T, Yagi N, Omatsu T, Hayashi S, Nakahata Y, Yasuda Y, Obora A, Kojima T, Naito Y, Itoh Y (2021). Benefits of linked color imaging for recognition of early differentiated-type gastric cancer: in comparison with indigo carmine contrast method and blue laser imaging. Surg Endosc.

[70] Yokoyama T, Miyahara R, Funasaka K, Furukawa K, Yamamura T, Ohno E, Nakamura M, Kawashima H, Watanabe O, Hirooka Y (2019). The utility of ultrathin endoscopy with flexible spectral imaging color enhancement for early gastric cancer. Nagoya J Med Sci.

[71] Osawa H, Yamamoto H, Miura Y, Ajibe H, Shinhata H, Yoshizawa M, Sunada K, Toma S, Satoh K, Sugano K (2012). Diagnosis of depressed-type early gastric cancer using small-caliber endoscopy with flexible spectral imaging color enhancement. Dig Endosc.

[72] Abe S, Makiguchi ME, Nonaka S, Suzuki H, Yoshinaga S, Saito Y (2022). Emerging texture and color enhancement imaging in early gastric cancer. Dig Endosc.

[73] Koyama Y, Sugimoto M, Kawai T, Mizumachi M, Yamanishi F, Matsumoto S, Suzuki Y, Nemoto D, Shinohara H, Ichimiya T (2023). Visibility of early gastric cancers by texture and color enhancement imaging using a high-definition ultrathin transnasal endoscope. Sci Rep.

[74] Sugimoto M, Kawai Y, Morino Y, Hamada M, Iwata E, Niikura R, Nagata N, Koyama Y, Fukuzawa M, Itoi T, Kawai T (2022). Efficacy of high-vision transnasal endoscopy using texture and colour enhancement imaging and narrow-band imaging to evaluate gastritis: a randomized controlled trial. Ann Med.

[75] Waki K, Kanesaka T, Michida T, Ishihara R, Tanaka Y (2022). Improved visibility of early gastric cancer by using a combination of chromoendoscopy and texture and color enhancement imaging. Gastrointest Endosc.

[76] Shijimaya T, Tahara T, Uragami T, Yano N, Tokutomi Y, Uwamori A, Nishimon S, Kobayashi S, Matsumoto Y, Nakamura N (2023). Usefulness of texture and color enhancement imaging (TXI) in early gastric cancer found after Helicobacter pylori eradication. Sci Rep.

[77] Kodashima S, Fujishiro M (2010). Novel image-enhanced endoscopy with i-scan technology. World J Gastroenterol.

[78] Nishimura J, Nishikawa J, Nakamura M, Goto A, Hamabe K, Hashimoto S, Okamoto T, Suenaga M, Fujita Y, Hamamoto Y, Sakaida I (2014). Efficacy of i-Scan Imaging for the Detection and Diagnosis of Early Gastric Carcinomas. Gastroenterol Res Pract.

[79] Koh M, Lee JY, Han SH, Jeon SW, Kim SJ, Cho JY, Kim SH, Jang JY, Baik GH, Jang JS (2023). Comparison Trial between I-SCAN-Optical Enhancement and Chromoendoscopy for Evaluating the Horizontal Margins of Gastric Epithelial Neoplasms. Gut Liver.

[80] Tosun Y, Velidedeoğlu M, Akıncı O, Ferahman S, Kepil N, Tortum OB (2022). Comparison of the effectiveness of i-scan and conventional endoscopy in the detection of the endoscopic signs of atrophic gastritis: A clinical trial. Arab J Gastroenterol.

[81] Sumiyama K (2017). Past and current trends in endoscopic diagnosis for early stage gastric cancer in Japan. Gastric Cancer.

[82] Abad MRA, Inoue H, Ikeda H, Manolakis A, Rodriguez de Santiago E, Sharma A, Fujiyoshi Y, Fukuda H, Sumi K, Onimaru M, Shimamura Y (2019). Utilizing fourth-generation endocytoscopy and the 'enlarged nuclear sign' for *in vivo* diagnosis of early gastric cancer. Endosc Int Open.

[83] Kaise M, Ohkura Y, Iizuka T, Kimura R, Nomura K, Kuribayashi Y, Yamada A, Yamashita S, Furuhata T, Kikuchi D (2015). Endocytoscopy is a promising modality with high diagnostic accuracy for gastric cancer. Endoscopy.

[84] Yan Y, Ma Z, Ji X, Liu J, Ji K, Li S, Wu Q (2022). A potential decision-making algorithm based on endoscopic ultrasound for staging early gastric cancer: a retrospective study. BMC Cancer.

[85] Kim SJ, Lim CH, Lee BI (2022). Accuracy of Endoscopic Ultrasonography for Determining the Depth of Invasion in Early Gastric Cancer. Turk J Gastroenterol.

[86] Pilonis ND, Januszewicz W, di Pietro M (2022). Confocal laser endomicroscopy in gastro-intestinal endoscopy: technical aspects and clinical applications. Transl Gastroenterol Hepatol.

[87] Zhou YW, Zhang LY, Ding SN, Zhang AL, Zhu Y, Chen YX, Zhang QC, Sun LT, Yu JR (2022). Hesitate between confocal laser endomicroscopy and narrow-band imaging: how to choose a better method in the detection of focal precancerous state of gastric cancer. Am J Transl Res.

[88] Li Y, Liu H, Huang H, Zhu Y, Deng H, Yu J, Luo S, Huo L, Lin L, Xie H, Li G (2017). [Progress of the application of optical coherence tomography in gastrointestinal tumor surgery]. Zhonghua Wei Chang Wai Ke Za Zhi.

[89] Liao Z, Zou W, Li ZS (2018). Clinical application of magnetically controlled capsule gastroscopy in gastric disease diagnosis: recent advances. Sci China Life Sci.

[90] Li Z, Liu J, Ji CR, Chen FX, Liu FG, Ge J, Chen Y, Sun XG, Lu YY, Cheng GH (2021). Screening for upper gastrointestinal cancers with magnetically controlled capsule gastroscopy: a feasibility study. Endoscopy.

[91] An P, Yang D, Wang J, Wu L, Zhou J, Zeng Z, Huang X, Xiao Y, Hu S, Chen Y (2020). A deep learning method for delineating early gastric cancer resection margin under chromoendoscopy and white light endoscopy. Gastric Cancer.

[92] Yuan XL, Zhou Y, Liu W, Luo Q, Zeng XH, Yi Z, Hu B (2022). Artificial intelligence for diagnosing gastric lesions under white-light endoscopy. Surg Endosc.

[93] Feng F, Tian Y, Xu G, Liu Z, Liu S, Zheng G, Guo M, Lian X, Fan D, Zhang H (2017). Diagnostic and prognostic value of CEA, CA19-9, AFP and CA125 for early gastric cancer. BMC Cancer.

[94] Shibata C, Nakano T, Yasumoto A, Mitamura A, Sawada K, Ogawa H, Miura T, Ise I, Takami K, Yamamoto K, Katayose Y (2022). Comparison of CEA and CA19-9 as a predictive factor for recurrence after curative gastrectomy in gastric cancer. BMC Surg.

[95] Yan J, Shao Y, Lu H, Ye Q, Ye G, Guo J (2022). Hsa_circ_0001020 Serves as a Potential Biomarker for Gastric Cancer Screening and Prognosis. Dig Dis Sci.

[96] Shimada H, Noie T, Ohashi M, Oba K, Takahashi Y (2014). Clinical significance of serum tumor markers for gastric cancer: a systematic review of literature by the Task Force of the Japanese Gastric Cancer Association. Gastric Cancer.

[97] Marrelli D, Pinto E, De Stefano A, Farnetani M, Garosi L, Roviello F (2001). Clinical utility of CEA, CA 19-9, and CA 72-4 in the follow-up of patients with resectable gastric cancer. Am J Surg.

[98] Xu Y, Zhang P, Zhang K, Huang C (2021). The application of CA72-4 in the diagnosis, prognosis, and treatment of gastric cancer. Biochim Biophys Acta Rev Cancer.

[99] Wang H, Jin W, Wan C, Zhu C (2022). Diagnostic value of combined detection of CA72-4, CA19-9, and carcinoembryonic antigen comparing to CA72-4 alone in gastric cancer: a systematic review and meta-analysis. Transl Cancer Res.

[100] Kodama I, Koufuji K, Kawabata S, Tetsu S, Tsuji Y, Takeda J, Kakegawa T (1995). The clinical efficacy of CA 72-4 as serum marker for gastric cancer in comparison with CA19-9 and CEA. Int Surg.

[101] Miao J, Liu Y, Zhao G, Liu X, Ma Y, Li H, Li S, Zhu Y, Xiong S, Zheng M, Fei S (2020). Feasibility of Plasma-Methylated SFRP2 for Early Detection of Gastric Cancer. Cancer Control.

[102] Shan M, Tian Q, Zhang L (2019). Serum CA50 levels in patients with cancers and other diseases. Prog Mol Biol Transl Sci.

[103] Xiao M, Zhang Z, Liao G, Liu M, Chen X (2022). Analysis of the Value of Helicobacter pylori Test in Combination with the Determination of Plasma Propepsin and Gastrin 17 in Screening the Precancerous Status of Gastric Cancer. Cell Mol Biol (Noisy-le-grand).

[104] Aoki K, Misumi J, Kimura T, Zhao W, Xie T (1997). Evaluation of cutoff levels for screening of gastric cancer using serum pepsinogens and distributions of levels of serum pepsinogen I, II and of PG I/PG II ratios in a gastric cancer case-control study. J Epidemiol.

[105] Samloff IM (1971). Cellular localization of group I pepsinogens in human gastric mucosa by immunofluorescence. Gastroenterology.

[106] Samloff IM, Varis K, Ihamaki T, Siurala M, Rotter JI (1982). Relationships among serum pepsinogen I, serum pepsinogen II, and gastric mucosal histology. A study in relatives of patients with pernicious anemia. Gastroenterology.

[107] Miki K, Ichinose M, Shimizu A, Huang SC, Oka H, Furihata C, Matsushima T, Takahashi K (1987). Serum pepsinogens as a screening test of extensive chronic gastritis. Gastroenterol Jpn.

[108] Kalniņa Z, Meistere I, Kikuste I, Tolmanis I, Zayakin P, Linē A (2015). Emerging blood-based biomarkers for detection of gastric cancer. World J Gastroenterol.

[109] Wang R, Chen XZ (2020). Prevalence of atrophic gastritis in southwest China and predictive strength of serum gastrin-17: A cross-sectional study (SIGES). Sci Rep.

[110] Liu W, Sun Y, Yuan Y (2020). Analysis of serum gastrin-17 and Helicobacter pylori antibody in healthy Chinese population. J Clin Lab Anal.

[111] Shen H, Xiong K, Wu X, Cheng S, Lou Q, Jin H, Zhang X (2021). The Diagnostic Value of Serum Gastrin-17 and Pepsinogen for Gastric Cancer Screening in Eastern China. Gastroenterol Res Pract.

[112] Ohata H, Kitauchi S, Yoshimura N, Mugitani K, Iwane M, Nakamura H, Yoshikawa A, Yanaoka K, Arii K, Tamai H (2004). Progression of chronic atrophic gastritis associated with Helicobacter pylori infection increases risk of gastric cancer. Int J Cancer.

[113] Yamaguchi Y, Nagata Y, Hiratsuka R, Kawase Y, Tominaga T, Takeuchi S, Sakagami S, Ishida S (2016). Gastric Cancer Screening by Combined Assay for Serum Anti-Helicobacter pylori IgG Antibody and Serum Pepsinogen Levels-The ABC Method. Digestion.

[114] Miki K (2011). Gastric cancer screening by combined assay for serum anti-Helicobacter pylori IgG antibody and serum pepsinogen levels - "ABC method". Proc Jpn Acad Ser B Phys Biol Sci.

[115] Watabe H, Mitsushima T, Yamaji Y, Okamoto M, Wada R, Kokubo T, Doi H, Yoshida H, Kawabe T, Omata M (2005). Predicting the development of gastric cancer from combining Helicobacter pylori antibodies and serum pepsinogen status: a prospective endoscopic cohort study. Gut.

[116] Kishikawa H, Kimura K, Takarabe S, Kaida S, Nishida J (2015). Helicobacter pylori Antibody Titer and Gastric Cancer Screening. Dis Markers.

[117] Yanaoka K, Oka M, Mukoubayashi C, Yoshimura N, Enomoto S, Iguchi M, Magari H, Utsunomiya H, Tamai H, Arii K (2008). Cancer high-risk subjects identified by serum pepsinogen tests: outcomes after 10-year follow-up in asymptomatic middle-aged males. Cancer Epidemiol Biomarkers Prev.

[118] Yanaoka K, Oka M, Yoshimura N, Mukoubayashi C, Enomoto S, Iguchi M, Magari H, Utsunomiya H, Tamai H, Arii K (2008). Risk of gastric cancer in asymptomatic, middle-aged Japanese subjects based on serum pepsinogen and Helicobacter pylori antibody levels. Int J Cancer.

[119] Li MY, Zhang DQ, Lu X, Chen WC (2018). [Comparison of two serological methods in screening gastric cancer and its precancerous condition]. Zhonghua Nei Ke Za Zhi.

[120] Ni DQ, Lyu B, Bao HB, Jin HF, Zhao J, Xu Y, Huang X (2019). [Comparison of different serological methods in screening early gastric cancer]. Zhonghua Nei Ke Za Zhi.

[121] Wang XT, Ji ZZ, Han F, Lyu B (2021). [A comparative study of new gastric cancer screening scoring system and new ABC method for screening gastric cancer and precancerous lesions]. Zhonghua Nei Ke Za Zhi.

[122] Zhang X, Hong L, Chan WY, Qiao T, Chen B, Liu Y, Fan D (2006). Expression of MG7-Ag in patients with gastric cancer correlates with weaker T cell immune response and more proinflammatory cytokine secretion. Biochem Cell Biol.

[123] Zhang L, Ren J, Pan K, Ma J, Li J, Shen L, Zhang X, Li J, Fan D, Gail M, You W (2010). Detection of gastric carcinoma-associated MG7-Ag by serum immuno-PCR assay in a high-risk Chinese population, with implication for screening. Int J Cancer.

[124] Hong L, Li S, Liu L, Shi Y, Wu K, Fan D (2010). The value of MG7-Ag and COX-2 for predicting malignancy in gastric precancerous lesions. Cell Biol Int.

[125] Wu S, Qu X, Wang N, Zhang L, Zhao X, Wu Q, Liu J, Shi Y (2023). MG7-Ag, hTERT, and TFF2 identified high-risk intestinal metaplasia and constituted a prediction model for gastric cancer. Chin Med J (Engl).

[126] Takahashi-Kanemitsu A, Knight CT, Hatakeyama M (2020). Molecular anatomy and pathogenic actions of Helicobacter pylori CagA that underpin gastric carcinogenesis. Cell Mol Immunol.

[127] Song L, Song M, Rabkin CS, Chung Y, Williams S, Torres J, Corvalan AH, Gonzalez R, Bellolio E, Shome M (2023). Identification of anti-Helicobacter pylori antibody signatures in gastric intestinal metaplasia. J Gastroenterol.

[128] Nishizawa T, Watanabe H, Yoshida S, Toyoshima A, Kataoka Y, Kanazawa T, Yoshizawa N, Ebinuma H, Suzuki H, Toyoshima O (2022). Decreased anti-parietal cell antibody titer in the advanced phase of autoimmune gastritis. Scand J Gastroenterol.

[129] Gao LM, Wang F, Zheng Y, Fu ZZ, Zheng L, Chen LL (2019). Roles of Fibroblast Activation Protein and Hepatocyte Growth Factor Expressions in Angiogenesis and Metastasis of Gastric Cancer. Pathol Oncol Res.

[130] Jelski W, Mroczko B (2022). Molecular and Circulating Biomarkers of Gastric Cancer. Int J Mol Sci.

[131] Preuss KD, Zwick C, Bormann C, Neumann F, Pfreundschuh M (2002). Analysis of the B-cell repertoire against antigens expressed by human neoplasms. Immunol Rev.

[132] Zayakin P, Ancāns G, Siliņa K, Meistere I, Kalniņa Z, Andrejeva D, Endzeliņš E, Ivanova L, Pismennaja A, Ruskule A (2013). Tumor-associated autoantibody signature for the early detection of gastric cancer. Int J Cancer.

[133] Zhou SL, Ku JW, Fan ZM, Yue WB, Du F, Zhou YF, Liu YL, Li Y, Tang S, Hu YL (2015). Detection of autoantibodies to a panel of tumor-associated antigens for the diagnosis values of gastric cardia adenocarcinoma. Dis Esophagus.

[134] Wang S, Qin J, Ye H, Wang K, Shi J, Ma Y, Duan Y, Song C, Wang X, Dai L (2018). Using a panel of multiple tumor-associated antigens to enhance autoantibody detection for immunodiagnosis of gastric cancer. Oncoimmunology.

[135] Pan Y, Zheng Y, Yang J, Wei Y, Wu H, Liu S, Yin A, Hu J, Zeng Y (2022). A new biomarker for the early diagnosis of gastric cancer: gastric juice- and serum-derived SNCG. Future Oncol.

[136] Zan X, Chen Z, Guo Q, Wang Y, Zhang Z, Ji R, Zheng Y, Zhang J, Wu Z, Li M (2022). The Association of Trefoil Factors with Gastric Cancer and Premalignant Lesions: A Cross-Sectional Population-Based Cohort Study. Cancer Epidemiol Biomarkers Prev.

[137] Liu CT, Wu FC, Zhuang YX, Huang XY, Li XH, Qu QQ, Peng YH, Xu YW, Chen SL, Huang XC (2023). The diagnostic value of serum insulin-like growth factor binding protein 7 in gastric cancer. PeerJ.

[138] Murphy G, Kamangar F, Dawsey SM, Stanczyk FZ, Weinstein SJ, Taylor PR, Virtamo J, Abnet CC, Albanes D, Freedman ND (2011). The relationship between serum ghrelin and the risk of gastric and esophagogastric junctional adenocarcinomas. J Natl Cancer Inst.

[139] Necula L, Matei L, Dragu D, Neagu AI, Mambet C, Nedeianu S, Bleotu C, Diaconu CC, Chivu-Economescu M (2019). Recent advances in gastric cancer early diagnosis. World J Gastroenterol.

[140] Kolesnikova EV, Tamkovich SN, Bryzgunova OE, Shelestyuk PI, Permyakova VI, Vlassov VV, Tuzikov AS, Laktionov PP, Rykova EY (2008). Circulating DNA in the blood of gastric cancer patients. Ann N Y Acad Sci.

[141] Wu R, Shi C, Chen Q, Wu F, Li Q (2020). Detection of circulating tumor cell DNA for monitoring advanced gastric cancer. Int J Clin Exp Pathol.

[142] Qian C, Ju S, Qi J, Zhao J, Shen X, Jing R, Yu J, Li L, Shi Y, Zhang L (2017). Alu-based cell-free DNA: a novel biomarker for screening of gastric cancer. Oncotarget.

[143] Sumbal S, Javed A, Afroze B, Zulfiqar HF, Javed F, Noreen S, Ijaz B (2018). Circulating tumor DNA in blood: Future genomic biomarkers for cancer detection. Exp Hematol.

[144] Kim K, Shin DG, Park MK, Baik SH, Kim TH, Kim S, Lee S (2014). Circulating cell-free DNA as a promising biomarker in patients with gastric cancer: diagnostic validity and significant reduction of cfDNA after surgical resection. Ann Surg Treat Res.

[145] Jung KW, Won YJ, Kong HJ, Lee ES (2018). Cancer Statistics in Korea: Incidence, Mortality, Survival, and Prevalence in 2015. Cancer Res Treat.

[146] Park JL, Kim HJ, Choi BY, Lee HC, Jang HR, Song KS, Noh SM, Kim SY, Han DS, Kim YS (2012). Quantitative analysis of cell-free DNA in the plasma of gastric cancer patients. Oncol Lett.

[147] Ren J, Lu P, Zhou X, Liao Y, Liu X, Li J, Wang W, Wang J, Wen L, Fu W, Tang F (2022). Genome-Scale Methylation Analysis of Circulating Cell-Free DNA in Gastric Cancer Patients. Clin Chem.

[148] Guo L, Huang C, Ji QJ (2017). Aberrant promoter hypermethylation of p16, survivin, and retinoblastoma in gastric cancer. Bratisl Lek Listy.

[149] Pimson C, Ekalaksananan T, Pientong C, Promthet S, Putthanachote N, Suwanrungruang K, Wiangnon S (2016). Aberrant methylation of PCDH10 and RASSF1A genes in blood samples for non-invasive diagnosis and prognostic assessment of gastric cancer. PeerJ.

[150] van der Vaart M, Pretorius PJ (2010). Is the role of circulating DNA as a biomarker of cancer being prematurely overrated?. Clin Biochem.

[151] Kim DG, An JY, Kim H, Shin SJ, Choi S, Seo WJ, Roh CK, Cho M, Son T, Kim HI (2019). Clinical Implications of Microsatellite Instability in Early Gastric Cancer. J Gastric Cancer.

[152] Comprehensive molecular characterization of gastric adenocarcinoma Nature. 2014;513(7517):202-9.

[153] Puliga E, Corso S, Pietrantonio F, Giordano S (2021). Microsatellite instability in Gastric Cancer: Between lights and shadows. Cancer Treat Rev.

[154] Chao J, Fuchs CS, Shitara K, Tabernero J, Muro K, Van Cutsem E, Bang YJ, De Vita F, Landers G, Yen CJ (2021). Assessment of Pembrolizumab Therapy for the Treatment of Microsatellite Instability-High Gastric or Gastroesophageal Junction Cancer Among Patients in the KEYNOTE-059, KEYNOTE-061, and KEYNOTE-062 Clinical Trials. JAMA Oncol.

[155] Zepeda-Najar C, Palacios-Astudillo RX, Chávez-Hernández JD, Lino-Silva LS, Salcedo-Hernández RA (2021). Prognostic impact of microsatellite instability in gastric cancer. Contemp Oncol (Pozn).

[156] Lin S, Gregory RI (2015). MicroRNA biogenesis pathways in cancer. Nat Rev Cancer.

[157] Finnegan EF, Pasquinelli AE (2013). MicroRNA biogenesis: regulating the regulators. Crit Rev Biochem Mol Biol.

[158] Li Y, Sun H, Guan J, Ji T, Wang X (2019). Serum microRNA-381: A Potential Marker for Early Diagnosis of Gastric Cancer. Yonsei Med J.

[159] ZiaSarabi P, Sorayayi S, Hesari A, Ghasemi F (2019). Circulating microRNA-133, microRNA-17 and microRNA-25 in serum and its potential diagnostic value in gastric cancer. J Cell Biochem.

[160] Kong Y, Ning L, Qiu F, Yu Q, Cao B (2019). Clinical significance of serum miR-25 as a diagnostic and prognostic biomarker in human gastric cancer. Cancer Biomark.

[161] Tsai MM, Wang CS, Tsai CY, Huang CG, Lee KF, Huang HW, Lin YH, Chi HC, Kuo LM, Lu PH, Lin KH (2016). Circulating microRNA-196a/b are novel biomarkers associated with metastatic gastric cancer. Eur J Cancer.

[162] Jiang Z, Guo J, Xiao B, Miao Y, Huang R, Li D, Zhang Y (2010). Increased expression of miR-421 in human gastric carcinoma and its clinical association. J Gastroenterol.

[163] Deng H, Guo Y, Song H, Xiao B, Sun W, Liu Z, Yu X, Xia T, Cui L, Guo J (2013). MicroRNA-195 and microRNA-378 mediate tumor growth suppression by epigenetical regulation in gastric cancer. Gene.

[164] Izumi D, Zhu Z, Chen Y, Toden S, Huo X, Kanda M, Ishimoto T, Gu D, Tan M, Kodera Y (2021). Assessment of the Diagnostic Efficiency of a Liquid Biopsy Assay for Early Detection of Gastric Cancer. JAMA Netw Open.

[165] Abe S, Matsuzaki J, Sudo K, Oda I, Katai H, Kato K, Takizawa S, Sakamoto H, Takeshita F, Niida S (2021). A novel combination of serum microRNAs for the detection of early gastric cancer. Gastric Cancer.

[166] Zhu C, Ren C, Han J, Ding Y, Du J, Dai N, Dai J, Ma H, Hu Z, Shen H (2014). A five-microRNA panel in plasma was identified as potential biomarker for early detection of gastric cancer. Br J Cancer.

[167] Huang Z, Zhu D, Wu L, He M, Zhou X, Zhang L, Zhang H, Wang W, Zhu J, Cheng W (2017). Six Serum-Based miRNAs as Potential Diagnostic Biomarkers for Gastric Cancer. Cancer Epidemiol Biomarkers Prev.

[168] Rathinasamy B, Velmurugan BK (2018). Role of lncRNAs in the cancer development and progression and their regulation by various phytochemicals. Biomed Pharmacother.

[169] Yao RW, Wang Y, Chen LL (2019). Cellular functions of long noncoding RNAs. Nat Cell Biol.

[170] Kopp F, Mendell JT (2018). Functional Classification and Experimental Dissection of Long Noncoding RNAs. Cell.

[171] Guo X, Peng Y, Song Q, Wei J, Wang X, Ru Y, Xu S, Cheng X, Li X, Wu D (2023). A Liquid Biopsy Signature for the Early Detection of Gastric Cancer in Patients. Gastroenterology.

[172] Qin S, Yang L, Kong S, Xu Y, Liang B, Ju S (2021). LncRNA HCP5: A Potential Biomarker for Diagnosing Gastric Cancer. Front Oncol.

[173] Shao Y, Ye M, Li Q, Sun W, Ye G, Zhang X, Yang Y, Xiao B, Guo J (2016). LncRNA-RMRP promotes carcinogenesis by acting as a miR-206 sponge and is used as a novel biomarker for gastric cancer. Oncotarget.

[174] Tang X, Yu L, Bao J, Jiang P, Yan F (2019). Function of Long Noncoding RNA UCA1 on Gastric Cancer Cells and its Clinicopathological Significance in Plasma. Clin Lab.

[175] Zhang T, Piao HY, Guo S, Zhao Y, Wang Y, Zheng ZC, Zhang J (2020). LncRNA PCGEM1 enhances metastasis and gastric cancer invasion through targeting of miR-129-5p to regulate P4HA2 expression. Exp Mol Pathol.

[176] Dong L, Qi P, Xu MD, Ni SJ, Huang D, Xu QH, Weng WW, Tan C, Sheng WQ, Zhou XY, Du X (2015). Circulating CUDR, LSINCT-5 and PTENP1 long noncoding RNAs in sera distinguish patients with gastric cancer from healthy controls. Int J Cancer.

[177] Chen L, Ge C, Feng X, Fu H, Wang S, Zhu J, Linghu E, Zheng X (2022). Identification of Combinations of Plasma lncRNAs and mRNAs as Potential Biomarkers for Precursor Lesions and Early Gastric Cancer. J Oncol.

[178] Tao X, Shao Y, Yan J, Yang L, Ye Q, Wang Q, Lu R, Guo J (2021). Biological roles and potential clinical values of circular RNAs in gastrointestinal malignancies. Cancer Biol Med.

[179] Shao Y, Qi C, Yan J, Lu R, Ye G, Guo J (2022). Biological and clinical implications of hsa_circ_0086720 in gastric cancer and its clinical application. J Clin Lab Anal.

[180] Tang W, Fu K, Sun H, Rong D, Wang H, Cao H (2018). CircRNA microarray profiling identifies a novel circulating biomarker for detection of gastric cancer. Mol Cancer.

[181] Tao X, Shao Y, Lu R, Ye Q, Xiao B, Ye G, Guo J (2020). Clinical significance of hsa_circ_0000419 in gastric cancer screening and prognosis estimation. Pathol Res Pract.

[182] Wei J, Wei W, Xu H, Wang Z, Gao W, Wang T, Zheng Q, Shu Y, De W (2020). Circular RNA hsa_circRNA_102958 may serve as a diagnostic marker for gastric cancer. Cancer Biomark.

[183] Ye Q, Qi C, Xi M, Ye G (2021). Circular RNA hsa_circ_0001874 is an indicator for gastric cancer. J Clin Lab Anal.

[184] Li S, Han L (2019). Circular RNAs as promising biomarkers in cancer: detection, function, and beyond. Genome Med.

[185] Roy S, Kanda M, Nomura S, Zhu Z, Toiyama Y, Taketomi A, Goldenring J, Baba H, Kodera Y, Goel A (2022). Diagnostic efficacy of circular RNAs as noninvasive, liquid biopsy biomarkers for early detection of gastric cancer. Mol Cancer.

[186] Doyle LM, Wang MZ (2019). Overview of Extracellular Vesicles, Their Origin, Composition, Purpose, and Methods for Exosome Isolation and Analysis. Cells.

[187] Shi Y, Wang Z, Zhu X, Chen L, Ma Y, Wang J, Yang X, Liu Z (2020). Exosomal miR-1246 in serum as a potential biomarker for early diagnosis of gastric cancer. Int J Clin Oncol.

[188] Guo X, Lv X, Ru Y, Zhou F, Wang N, Xi H, Zhang K, Li J, Chang R, Xie T (2020). Circulating Exosomal Gastric Cancer-Associated Long Noncoding RNA1 as a Biomarker for Early Detection and Monitoring Progression of Gastric Cancer: A Multiphase Study. JAMA Surg.

[189] Xiao K, Li S, Ding J, Wang Z, Wang D, Cao X, Zhang Y, Dong Z (2022). Expression and clinical value of circRNAs in serum extracellular vesicles for gastric cancer. Front Oncol.

[190] Thanh Huong P, Gurshaney S, Thanh Binh N, Gia Pham A, Hoang Nguyen H, Thanh Nguyen X, Pham-The H, Tran PT, Truong Vu K, Xuan Duong N (2020). Emerging Role of Circulating Tumor Cells in Gastric Cancer. Cancers (Basel).

[191] Kang HM, Kim GH, Jeon HK, Kim DH, Jeon TY, Park DY, Jeong H, Chun WJ, Kim MH, Park J (2017). Circulating tumor cells detected by lab-on-a-disc: Role in early diagnosis of gastric cancer. PLoS One.

[192] Cheng R, Peng Y, Sun X, Zhang S, Li P (2023). Circulating Tumor Cells as Diagnostic Markers of Early Gastric Cancer and Gastric Precancerous Lesions. Oncology.

[193] Fang T, Wang Y, Yin X, Zhai Z, Zhang Y, Yang Y, You Q, Li Z, Ma Y, Li C (2020). Diagnostic Sensitivity of NLR and PLR in Early Diagnosis of Gastric Cancer. J Immunol Res.

[194] Kwak MH, Yang SM, Yun SK, Kim S, Choi MG, Park JM (2021). Identification and validation of LGR5-binding peptide for molecular imaging of gastric cancer. Biochem Biophys Res Commun.

[195] Shen Y, Xie Y, Yu X, Zhang S, Wen Q, Ye G, Guo J (2021). Clinical diagnostic values of transfer RNA-derived fragment tRF-19-3L7L73JD and its effects on the growth of gastric cancer cells. J Cancer.

[196] Shen Y, Yu X, Ruan Y, Li Z, Xie Y, Yan Z, Guo J (2021). Global profile of tRNA-derived small RNAs in gastric cancer patient plasma and identification of tRF-33-P4R8YP9LON4VDP as a new tumor suppressor. Int J Med Sci.

[197] Zhu L, Li Z, Yu X, Ruan Y, Shen Y, Shao Y, Zhang X, Ye G, Guo J (2021). The tRNA-derived fragment 5026a inhibits the proliferation of gastric cancer cells by regulating the PTEN/PI3K/AKT signaling pathway. Stem Cell Res Ther.

[198] Zhang S, Xie Y, Yu X, Ge J, Ye G, Guo J (2023). Absolute quantification of a plasma tRNA-derived fragment for the diagnosis and prognosis of gastric cancer. Front Oncol.

[199] Ohata H, Oka M, Yanaoka K, Shimizu Y, Mukoubayashi C, Mugitani K, Iwane M, Nakamura H, Tamai H, Arii K (2005). Gastric cancer screening of a high-risk population in Japan using serum pepsinogen and barium digital radiography. Cancer Sci.

